# Supplementary material for: MicroFinder: conserved gene-set mapping and assembly ordering for manual curation of bird dot microchromosomes
Source: Gigascience. 2026 Apr 3;15:giag036. doi: 10.1093/gigascience/giag036 (PMC13192246; doi:10.1093/gigascience/giag036)
Supplement: giag036_Supplemental_Files [file giag036_supplemental_files.zip › Supplementary Figures.pdf]

Supplementary Figures

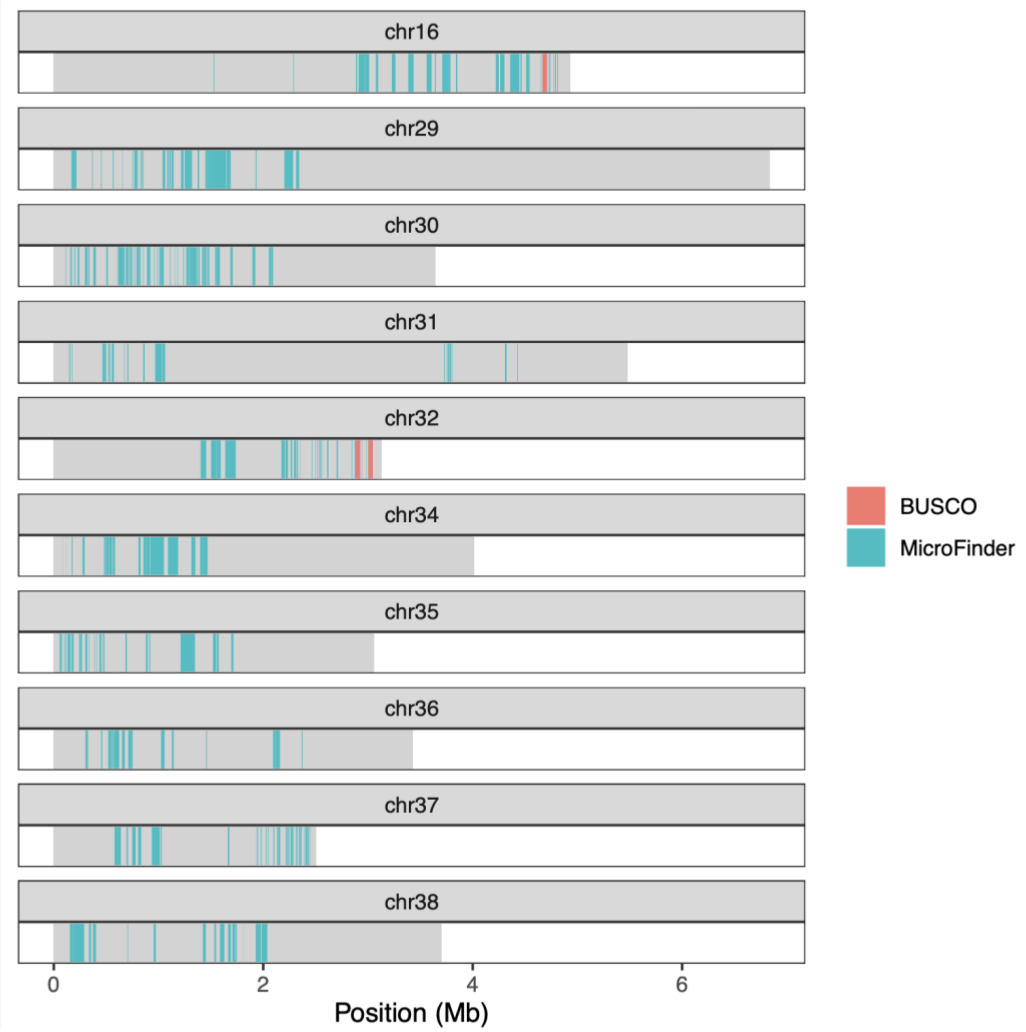

Supplementary Figure 1: Location of orthoDB10 avian BUSCOs (n = 3) and MicroFinder loci (n = 307) on chicken (GGswu assembly) dot chromosomes.

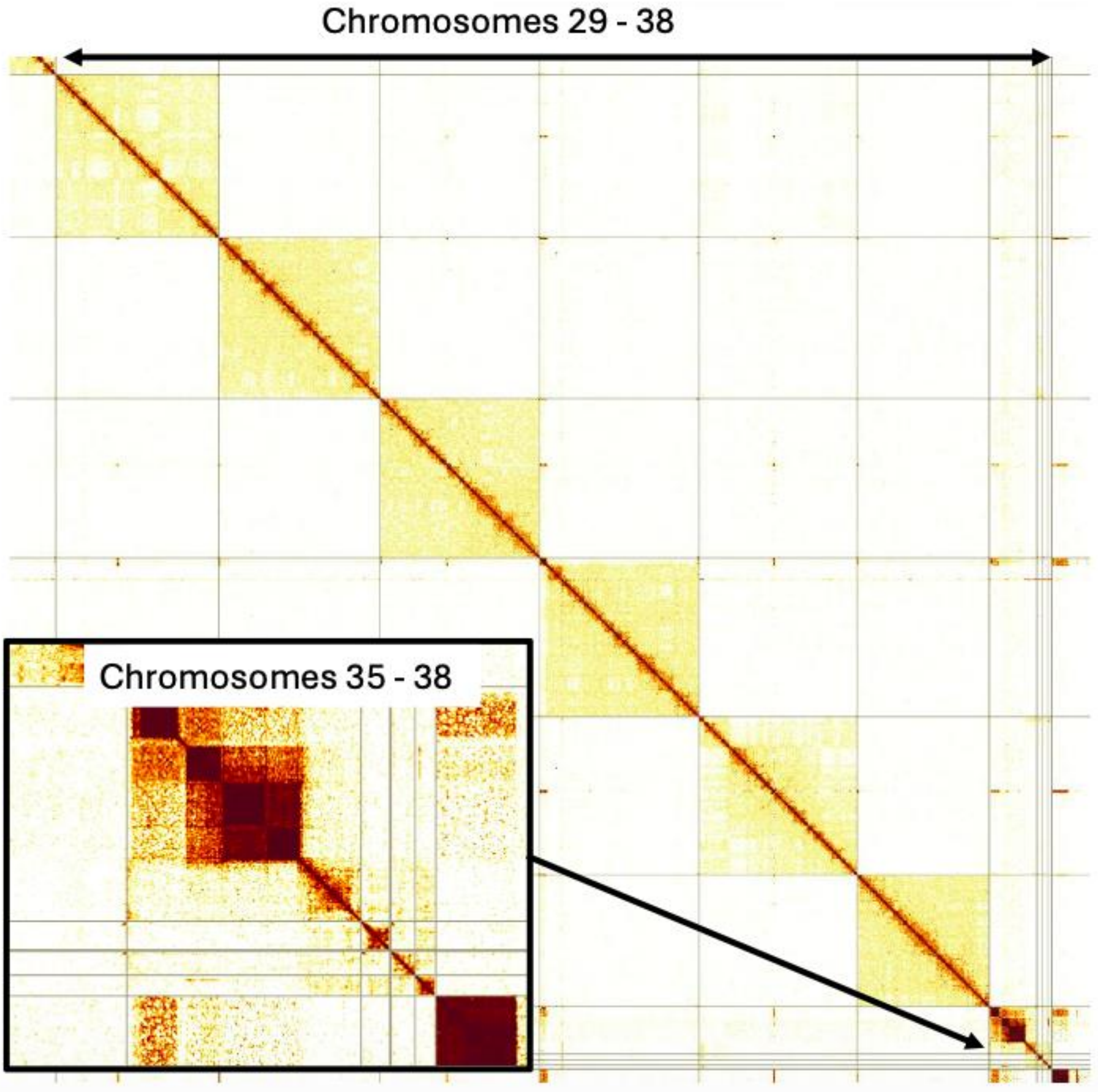

Supplementary Figure 2: HI-C contact map for the MicroFinder-curated assembly of *Accipiter gentilis* (bAccGen1 v3) showing the 10 smallest chromosomes. Grey vertical and horizontal lines demark boundaries between scaffolds in the assembly. Chromosomes are indicated above the contact map.

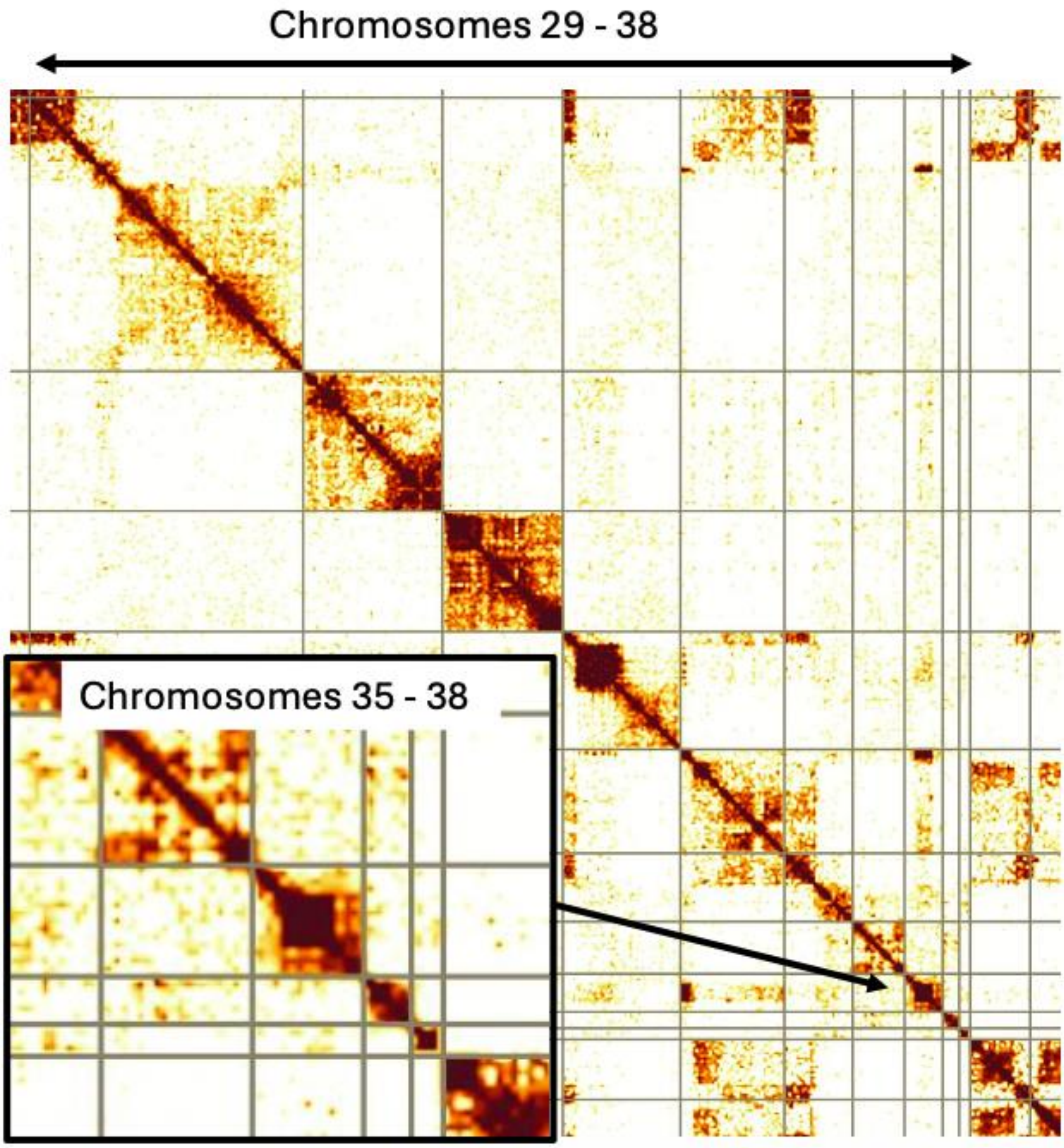

Supplementary Figure 3: HI-C contact map for the MicroFinder-curated assembly of *Anas acuta* (bAnaAcu1 v2) showing the 10 smallest chromosomes. Grey vertical and horizontal lines demark boundaries between scaffolds in the assembly. Chromosomes are indicated above the contact map.

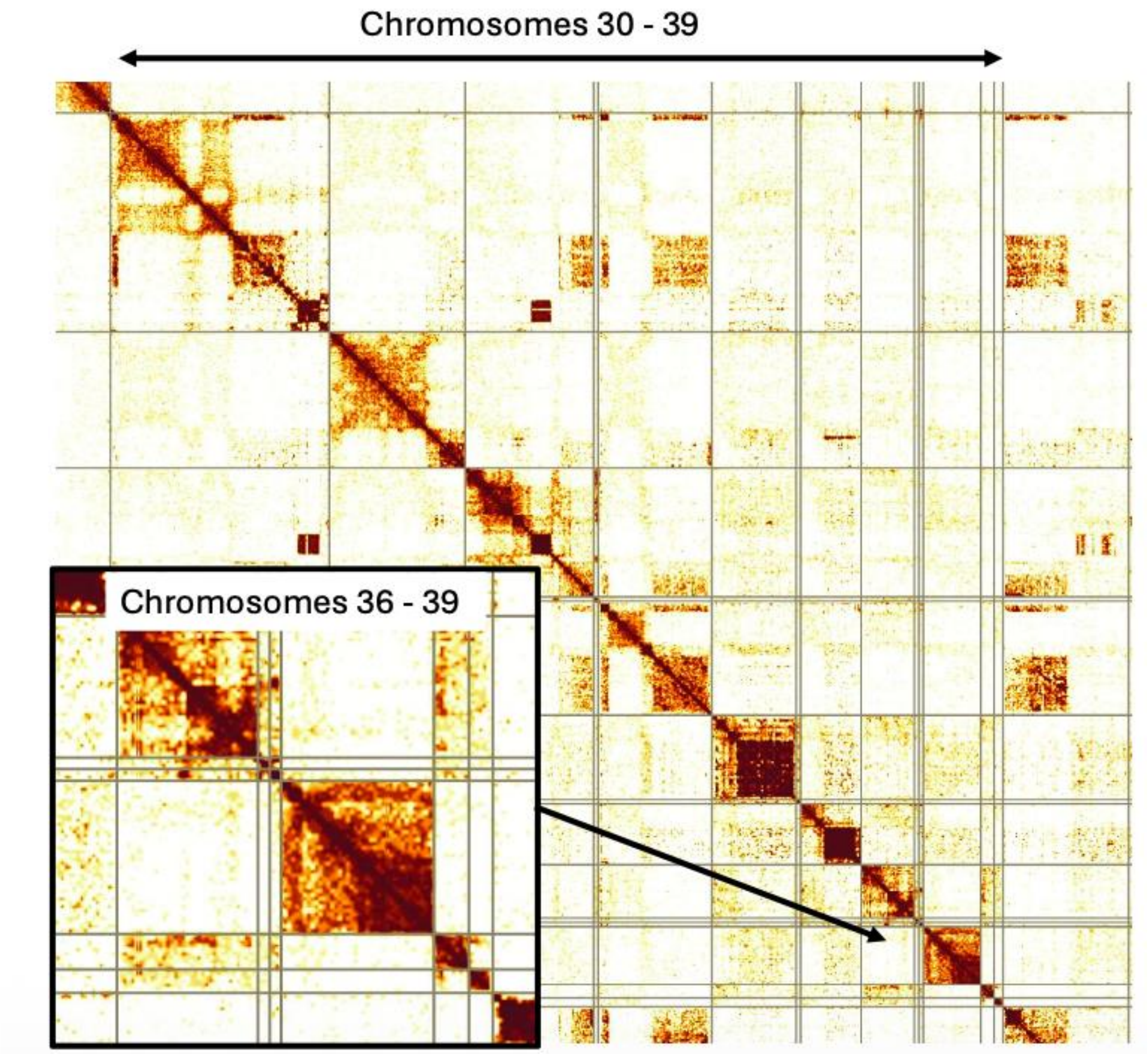

Supplementary Figure 4: HI-C contact map for the MicroFinder-curated assembly of *Anas platyrhynchos* (bAnaPla2 v2) showing the 10 smallest chromosomes. Grey vertical and horizontal lines demark boundaries between scaffolds in the assembly. Chromosomes are indicated above the contact map.

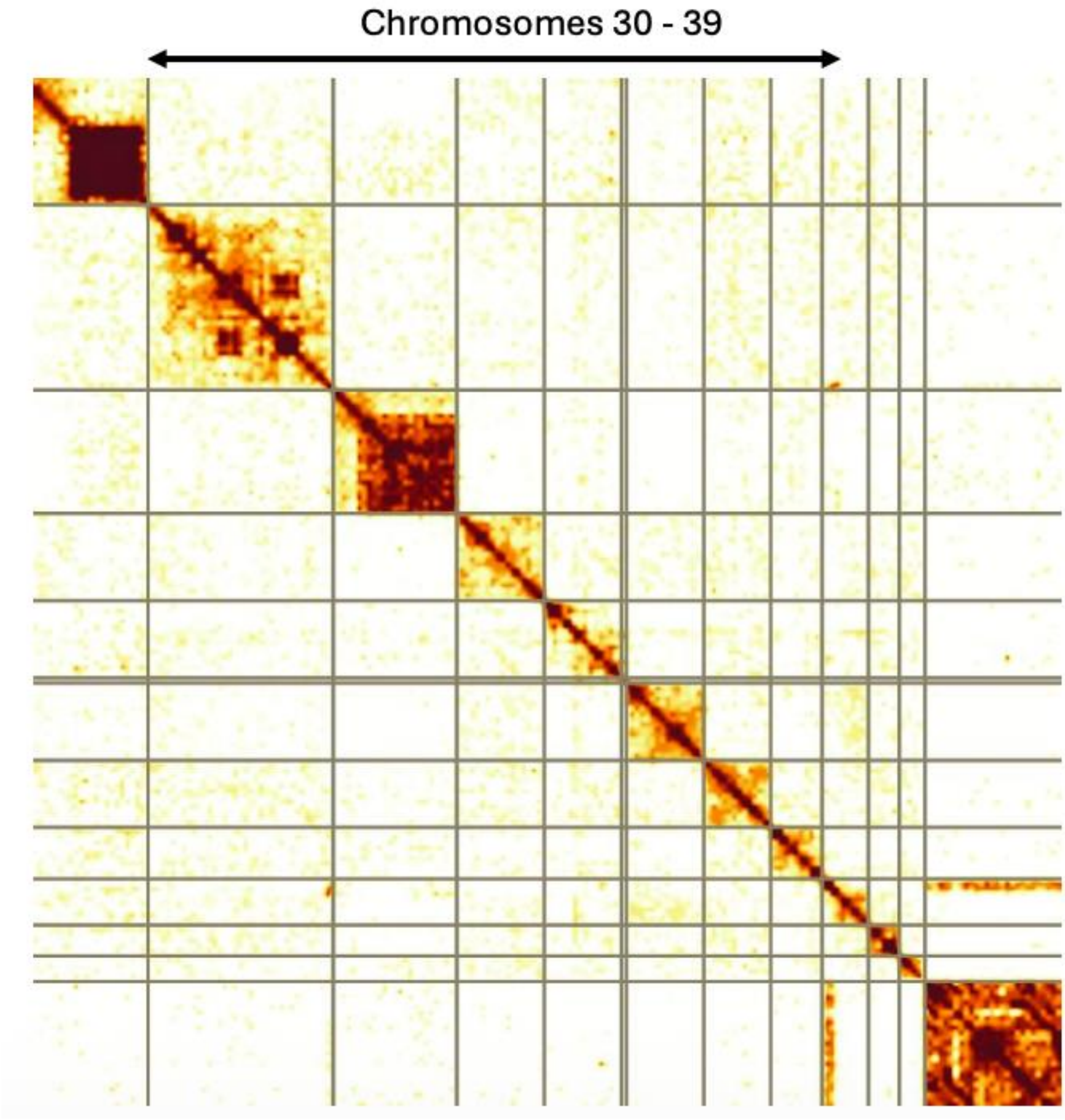

Supplementary Figure 5: HI-C contact map for the MicroFinder-curated assembly of *Anser brachyrhynchus* (bAnsBra1 v2) showing the 10 smallest chromosomes. Grey vertical and horizontal lines demark boundaries between scaffolds in the assembly. Chromosomes are indicated above the contact map.

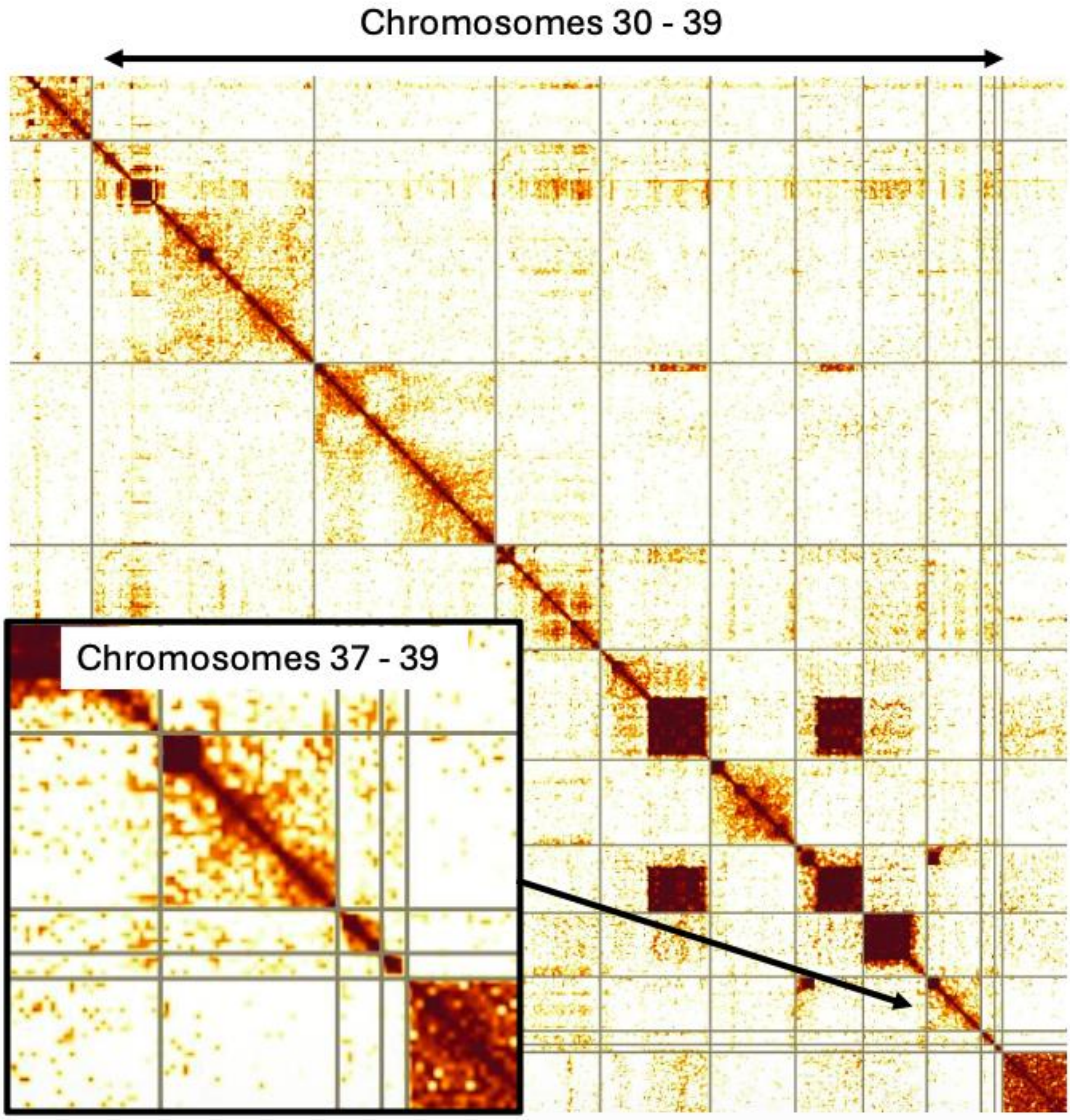

Supplementary Figure 6: HI-C contact map for the MicroFinder-curated assembly of *Bucephala clangula* (bBucCla1 v2) showing the 10 smallest chromosomes. Grey vertical and horizontal lines demark boundaries between scaffolds in the assembly. Chromosomes are indicated above the contact map.

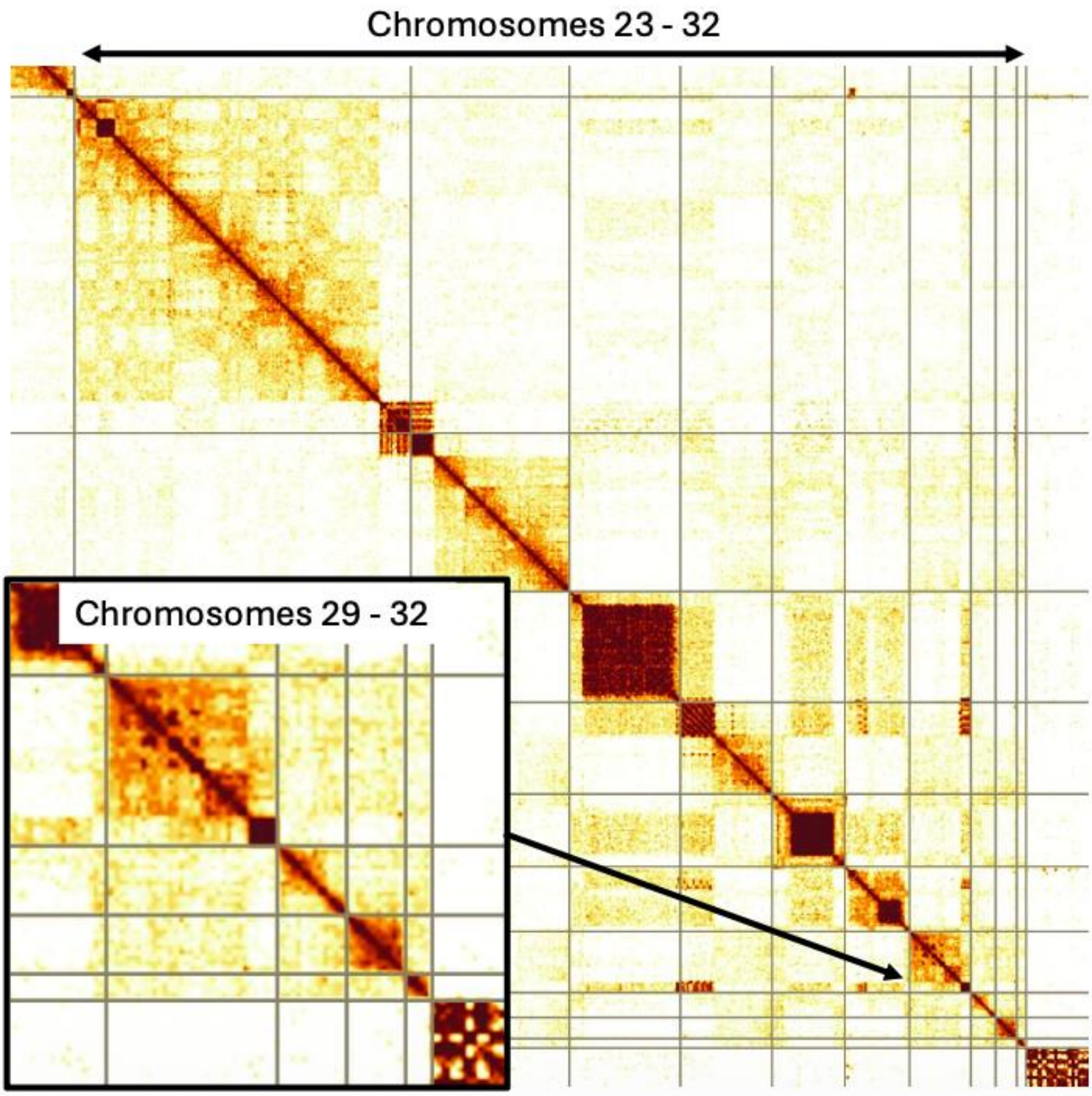

Supplementary Figure 7: HI-C contact map for the MicroFinder-curated assembly of *Chroicocephalus ridibundus* (bChrRid1 v2) showing the 10 smallest chromosomes. Grey vertical and horizontal lines demark boundaries between scaffolds in the assembly. Chromosomes are indicated above the contact map.

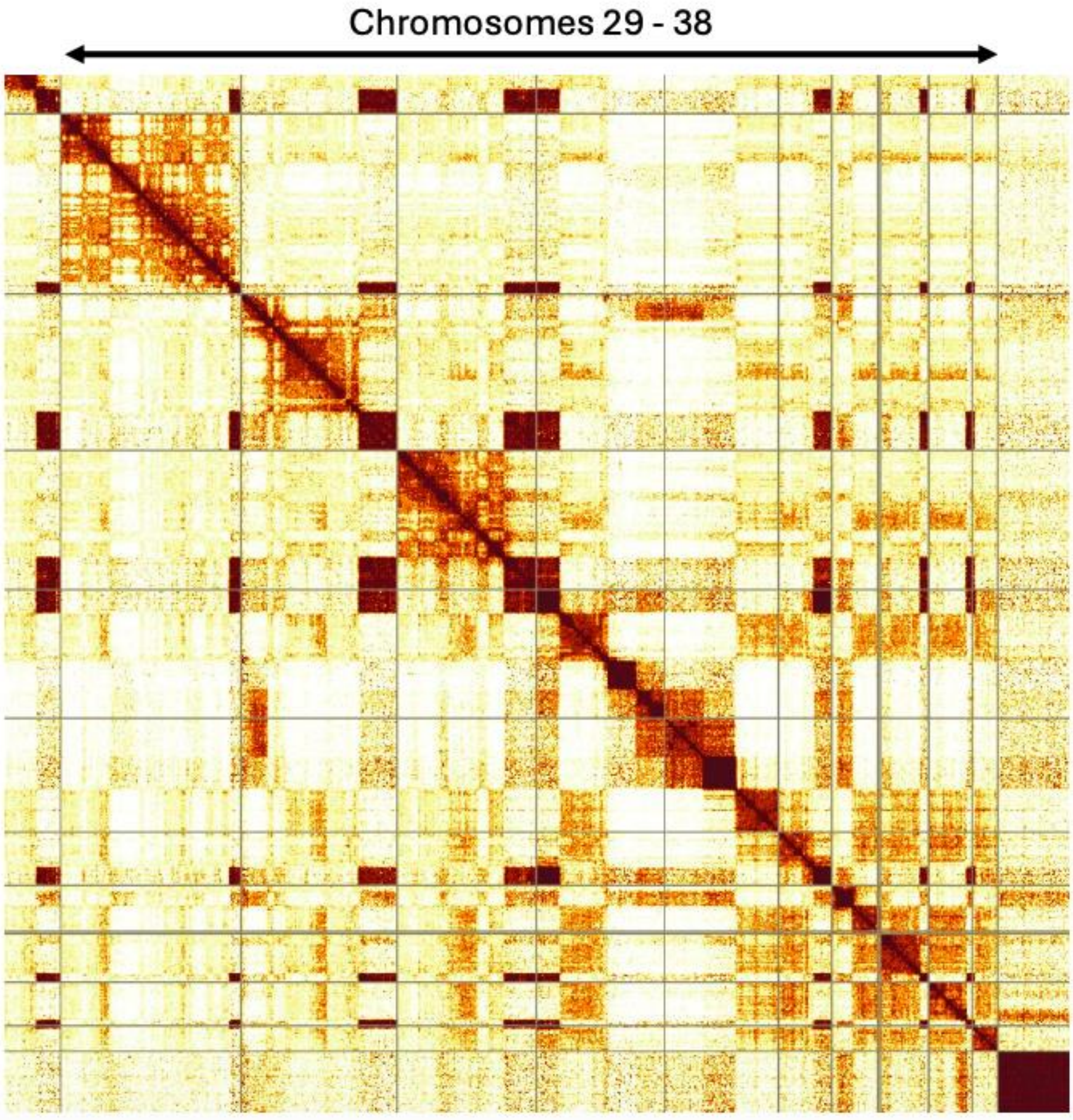

Supplementary Figure 8: HI-C contact map for the MicroFinder-curated assembly of *Cinclus cinclus* (bCinCin1 v2) showing the 10 smallest chromosomes. Grey vertical and horizontal lines demark boundaries between scaffolds in the assembly. Chromosomes are indicated above the contact map.

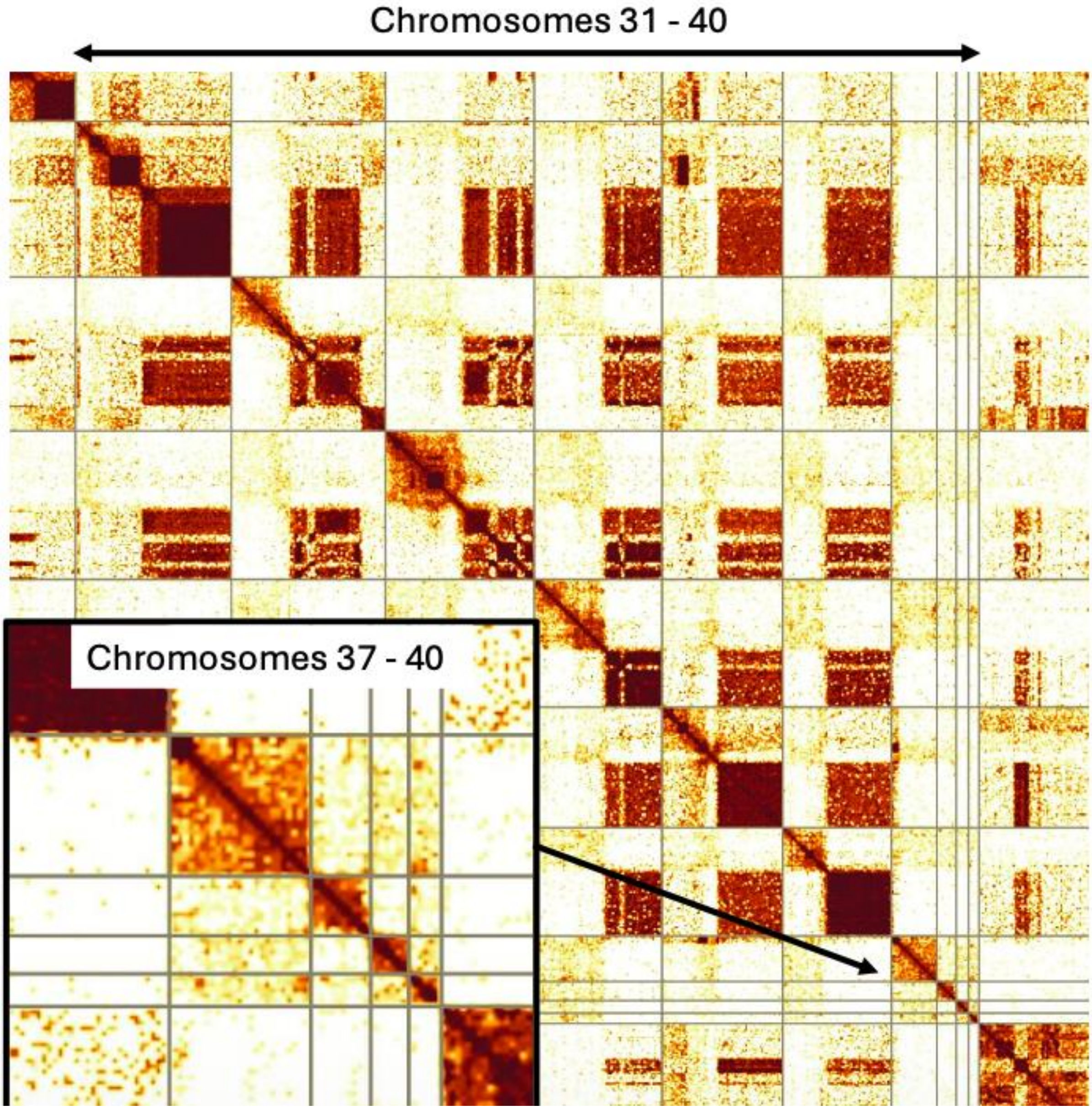

Supplementary Figure 9: HI-C contact map for the MicroFinder-curated assembly of *Clangula hyemalis* (bClaHye2 v2) showing the 10 smallest chromosomes. Grey vertical and horizontal lines demark boundaries between scaffolds in the assembly. Chromosomes are indicated above the contact map.

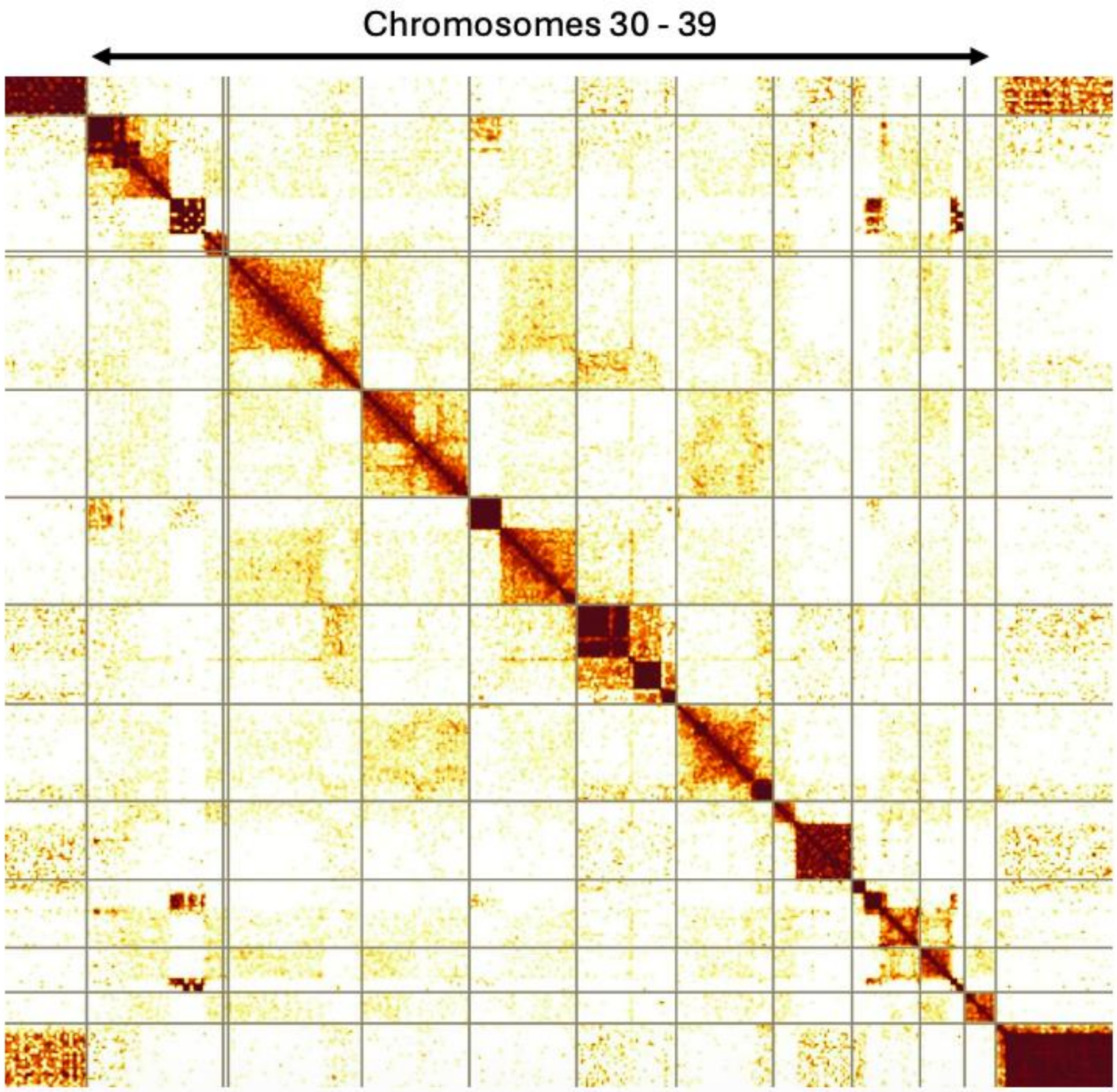

Supplementary Figure 10: HI-C contact map for the MicroFinder-curated assembly of *Grus grus* (bGruGru1 v2) showing the 10 smallest chromosomes. Grey vertical and horizontal lines demark boundaries between scaffolds in the assembly. Chromosomes are indicated above the contact map.

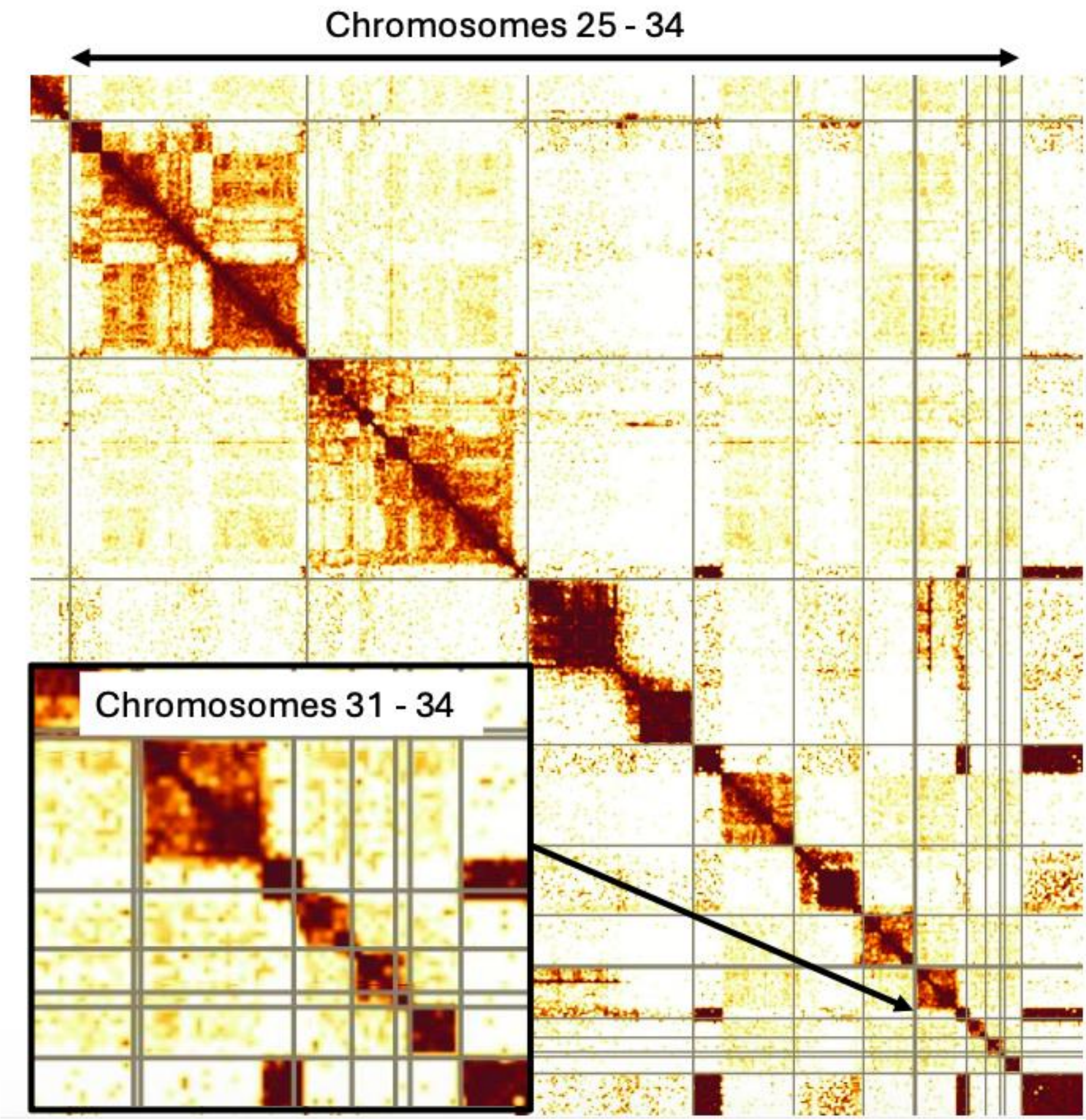

Supplementary Figure 11: HI-C contact map for the MicroFinder-curated assembly of *Gulosus aristotelis* (bGulAri2 v2) showing the 10 smallest chromosomes. Grey vertical and horizontal lines demark boundaries between scaffolds in the assembly. Chromosomes are indicated above the contact map.

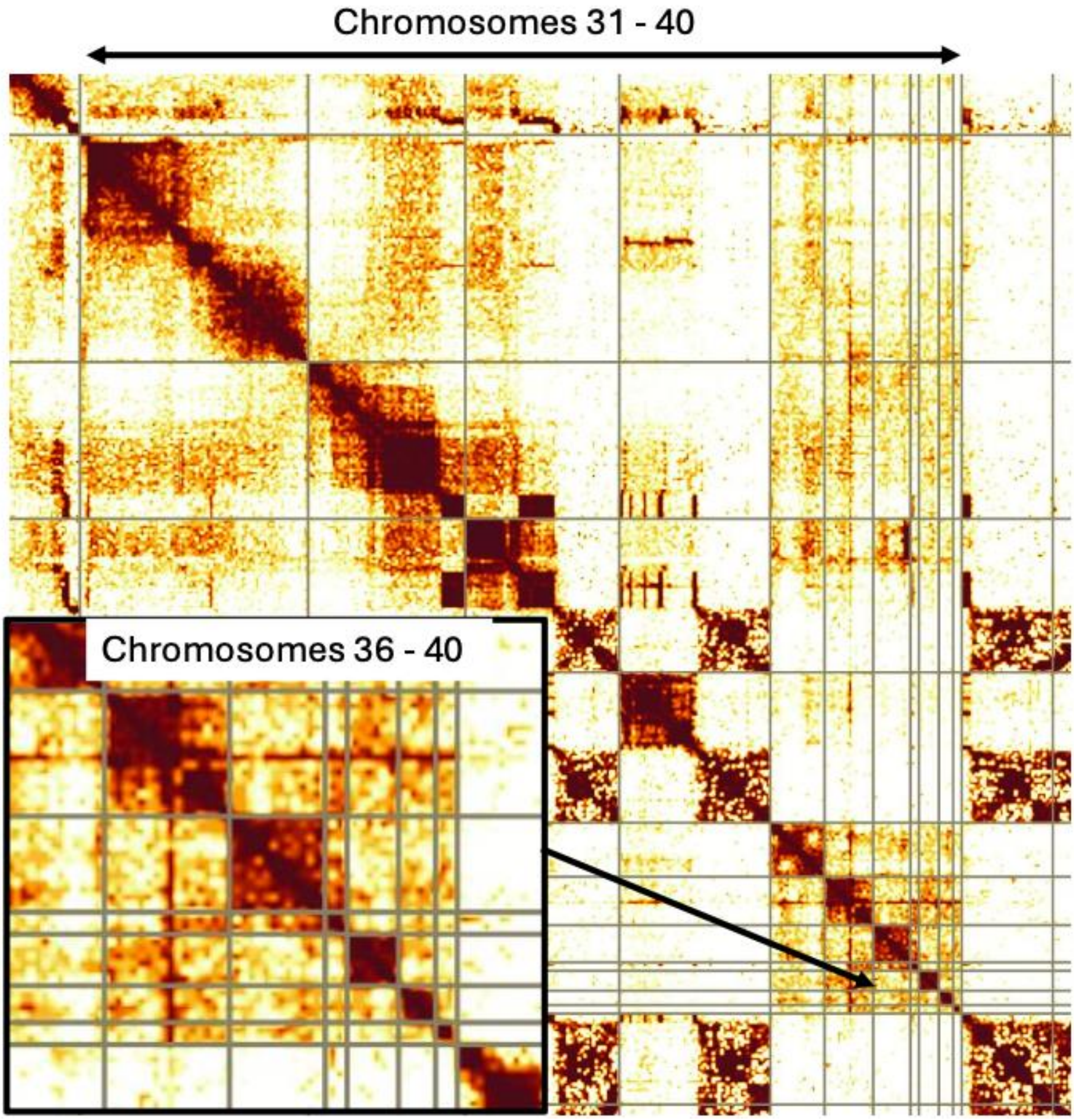

Supplementary Figure 12: HI-C contact map for the MicroFinder-curated assembly of *Netta rufina* (bNetRuf1 v2) showing the 10 smallest chromosomes. Grey vertical and horizontal lines demark boundaries between scaffolds in the assembly. Chromosomes are indicated above the contact map.

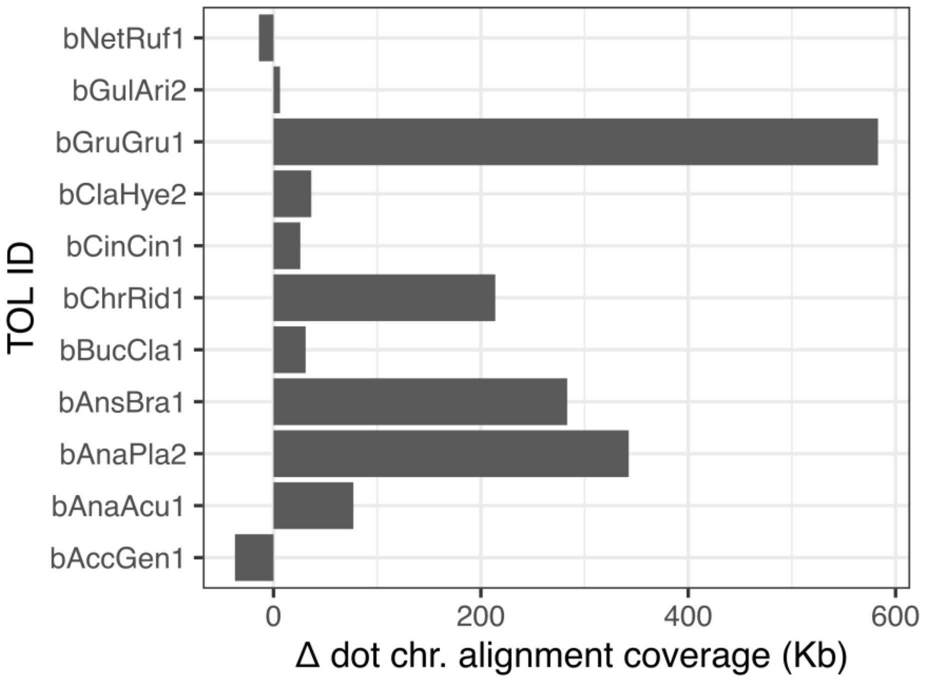

Supplementary Figure 13: Change in chicken (GGswu assembly) dot chromosome alignment coverage of chromosomally placed assembly content after MicroFinder-enabled manual curation for 11 DToL bird genome assemblies. Each DToL assembly was aligned to the GGswu chicken assembly before and after MicroFinder-enabled re-curation and the difference in the total number of covered bases was calculated. Only alignments involving chromosomally placed assembly content in the DToL assemblies were retained.
