# Supplementary material for: MicroFinder: conserved gene-set mapping and assembly ordering for manual curation of bird dot microchromosomes
Source: Gigascience. 2026 Apr 3;15:giag036. doi: 10.1093/gigascience/giag036 (PMC13192246; doi:10.1093/gigascience/giag036)
Supplement: giag036_GIGA-D-25-00217_revision_1 [file giag036_giga-d-25-00217_revision_1.pdf]

// GigaScience

GigaScience

MicroFinder: conserved gene-set mapping and assembly ordering for manual curation of bird microchromosomes

--Manuscript Draft--

**Manuscript Number:**	GIGA-D-25-00217R1
**Full Title:**	MicroFinder: conserved gene-set mapping and assembly ordering for manual curation of bird microchromosomes
**Article Type:**	Technical Note
**Funding Information:**	Wellcome Trust (220540) — Not applicable<br>Wellcome Trust (218328) — Not applicable
**Abstract:**	Background<br>Obtaining chromosomally complete genome assemblies across the tree of life is an important goal of biodiversity genomics. However, some lineages remain recalcitrant to assembly. Birds present a substantial assembly challenge due to the presence of tiny microchromosomes that are often highly fragmented or even missing in draft genome assemblies. Bird genomes therefore require substantial expert manual curation effort via manipulation of genome-wide HI-C contact maps and many chromosome-level bird genome assemblies do not resolve the known karyotype.<br>Findings<br>Here, using a reference set of expert curated bird genomes, we have identified a set of conserved proteins for the smallest and hardest to assemble microchromosomes – the dot chromosomes – and developed MicroFinder, a pipeline that uses this protein set to find small dot microchromosome fragments in draft genome assemblies to act as anchors for manual curation. We demonstrate how MicroFinder can be used to improve the speed and accuracy of bird genome curation. Furthermore, we highlight the usefulness of MicroFinder by carrying out MicroFinder-enabled re-curation of 12 previously released chromosome-scale bird genome assemblies, increasing the sequence content of dot microchromosome models.<br>Conclusions<br>We present MicroFinder, a pipeline to identify and order putative dot microchromosome scaffolds in draft genome assemblies. MicroFinder is an effective aid for bird genome assembly that dramatically speeds up manual assembly curation and improves the accuracy and sequence content of bird dot microchromosomes, even enabling improvement to genome assemblies that have already undergone expert curation.
**Corresponding Author:**	Thomas Charles Mathers<br>Wellcome Sanger Institute<br>Cambridge, Cambridgeshire UNITED KINGDOM
**Corresponding Author Secondary Information:**	
**Corresponding Author's Institution:**	Wellcome Sanger Institute
**Corresponding Author's Secondary Institution:**	
**First Author:**	Thomas Charles Mathers
**First Author Secondary Information:**	
**Order of Authors:**	Thomas Charles Mathers<br>Michael Paulini<br>Cibele G. Sotero-Caio<br>Jonathan M. D. Wood
**Order of Authors Secondary Information:**	
**Response to Reviewers:**	Please see attached cover letter.

Powered by Editorial Manager® and ProduXion Manager® from Aries Systems Corporation

Additional Information:	
**Question**	**Response**
Are you submitting this manuscript to a special series or article collection?	No
**Experimental design and statistics**<br><br>Full details of the experimental design and statistical methods used should be given in the Methods section, as detailed in our Minimum Standards Reporting Checklist. Information essential to interpreting the data presented should be made available in the figure legends.<br><br>Have you included all the information requested in your manuscript?	Yes
**Resources**<br><br>A description of all resources used, including antibodies, cell lines, animals and software tools, with enough information to allow them to be uniquely identified, should be included in the Methods section. Authors are strongly encouraged to cite Research Resource Identifiers (RRIDs) for antibodies, model organisms and tools, where possible.<br><br>Have you included the information requested as detailed in our Minimum Standards Reporting Checklist?	Yes
**Availability of data and materials**<br><br>All datasets and code on which the conclusions of the paper rely must be either included in your submission or deposited in publicly available repositories (where available and ethically appropriate), referencing such data using a unique identifier in the references and in the "Availability of Data and Materials" section of your manuscript.	Yes

Have you have met the above requirement as detailed in our Minimum Standards Reporting Checklist?	
GigaScience has policies and guidelines in place for the use of generative AI-writing tools such as ChatGPT. If you have used such writing tools to assist with writing the manuscript this must be declared and cited in the text. Authors should not list AI-writing tools and other AI-assisted technologies as an author or co-author and should acknowledge that they are fully responsible for text generated or refined by AI-writing tools.<p><br><br>A summary of use (particularly in the introduction or among methods) needs to be included at the end of the paper, and the outputs should also be included as a supplementary file hosted in GigaDB or other open repositories. Please read our guidelines for more information. <p><br><br>By submitting to GigaScience, you are aware of the journal's AI-writing tools policy, and if you have declared use of such tools below, you have acknowledged this where appropriate in your manuscript and have made a summary of use and outputs available. <p><br><b>AI-assisted writing tools have been used in the preparation of this manuscript?	No

Manuscript
Click here to access/download;Manuscript;MicroFinder_gigascience_final.d

MicroFinder: conserved gene-set mapping and assembly ordering for manual curation of bird dot microchromosomes

Thomas C. Mathers[1*], Michael Paulini[1], Cibele G. Sotero-Caio[1] and Jonathan M. D. Wood[1]

[1] Tree of Life, Wellcome Sanger Institute, Wellcome Genome Campus, Hinxton, Cambridge, CB10 1SA, UK.

[*] Corresponding author. Email: tm18@sanger.ac.uk.

ORCIDs: Thomas C Mathers [0000-0002-8637-3515]; Michael Paulini [0000-0002-6968-2340]; Cibele G Sotero-Caio [0000-0002-3112-0000]; Jonathan M D Wood [0000-0002-7545-2162]

Abstract

Background

Obtaining chromosomally complete genome assemblies across the tree of life is an important goal of biodiversity genomics. However, some lineages remain recalcitrant to assembly. Birds present a substantial assembly challenge due to the presence of tiny microchromosomes that are often highly fragmented or even missing in draft genome assemblies. Bird genomes therefore require substantial expert manual curation effort via manipulation of genome-wide HI-C contact maps and many chromosome-level bird genome assemblies do not resolve the known karyotype.

Findings

Here, using a reference set of expert curated bird genomes, we have identified a set of conserved proteins for the smallest and hardest to assemble microchromosomes – the dot chromosomes – and developed MicroFinder, a pipeline that uses this protein set to find small dot microchromosome fragments in draft genome assemblies to act as anchors for manual curation. We demonstrate how MicroFinder can be used to improve the speed and accuracy of bird genome curation. Furthermore, we highlight the usefulness of MicroFinder by carrying out MicroFinder-enabled re-curation of 12 previously released chromosome-scale bird genome assemblies, increasing the sequence content of dot microchromosome models.

Conclusions

We present MicroFinder, a pipeline to identify and order putative dot microchromosome scaffolds in draft genome assemblies. MicroFinder is an effective aid for bird genome assembly

that dramatically speeds up manual assembly curation and improves the accuracy and sequence content of bird dot microchromosomes, even enabling improvement to genome assemblies that have already undergone expert curation.

Keywords

Aves, genome assembly, dot chromosomes, comparative genomics, manual curation, karyotype.

Introduction

Recent advances in sequencing technology have dramatically improved the quantity, quality and taxonomic breadth of reference genome assemblies across the tree of life [1–4]. Automated assembly of accurate long reads followed by scaffolding with high throughput *in vivo* chromatin conformation capture sequence data (HI-C) and manual curation [5] routinely results in genome assemblies that meet or exceed accepted gold standard metrics [6]. However, some lineages are recalcitrant to assembly and challenges remain to generate complete, chromosomally resolved genome assemblies for all taxa [7].

Within vertebrates, birds present a substantial assembly challenge due to the presence of tiny, hard to assemble, microchromosomes. Since early cytogenetic studies, it has been recognised that bird genomes typically contain six to eight pairs of large macrochromosomes and 31 to 33 pairs of small microchromosomes [8–10]. In chicken, macrochromosome size based on a near-T2T assembly ranges from 250 Mb to 30 Mb, and microchromosomes range from 23 Mb to 2.5 Mb [11]. Ten of the smallest microchromosomes (ranging in size from 6.8 to 2.5 Mb) are further categorised as “dot” chromosomes based on their minute size, morphology,

epigenetic landscape and extensive pericentromeric heterochromatin [11]. Once considered unimportant DNA fragments [12,13], cytogenetics and genomics have revealed that microchromosomes are highly conserved across avian evolution and contain many important and highly expressed housekeeping genes [14–16]. Furthermore, microchromosomes have distinct genetic and epigenetic features setting them apart from macrochromosomes: they are GC-biased, gene-rich, highly methylated, and have distinct spatial organisation in the centre of the nucleus [17–21].

Most recent bird genome assembly projects follow the Vertebrate Genome Project (VGP) assembly pipeline which uses accurate PacBio HiFi long reads for *de novo* assembly combined with HI-C data for long range scaffolding and phasing [22]. This pipeline produces assemblies with excellent contiguity and completeness statistics. However, these metrics do not fully capture the challenge of assembling the smallest bird chromosomes as they represent a small fraction of the total sequence content. Strikingly, despite high-quality sequence data, bird genome assemblies often do not fully resolve the known karyotype (**Figure 1a**; **Supplementary Table 1**). Of 105 species with chromosome-scale genome assemblies in International Nucleotide Sequence Database Collaboration (INSDC) databases that also have karyotype data, 62 (59 %) differ from the expected karyotype by 2 or more chromosomes, with the majority (57/62) having fewer chromosomes than expected. Primarily, this is due to failure to assemble and identify the full set of microchromosomes [23,24] and even in karyotype-resolved assemblies, microchromosomes, and particularly the dot microchromosomes, are often highly fragmented and can be incomplete [25]. Painstaking manual curation of bird genomes after *de novo* assembly and scaffolding is therefore an essential assembly step. For example, the HI-C contact map for the draft genome assembly of

the pink-footed goose *Anser brachyrhynchus* (assembled by the Darwin Tree of Life (DToL) project [26]) reveals 28 clear chromosomal elements (**Figure 1b**), yet closely related karyotyped geese all have 40 or 41 chromosomes [27,28]. Therefore, at least 12 chromosomes are expected to be among the unplaced small scaffolds and contigs located at the bottom right of the HI-C contact map which predominantly contains repetitive sequence (**Figure 1C**). To resolve the assembly, genome curators sift through the unplaced content to identify and assemble dot microchromosome fragments (**Figure 1d**). Techniques include making use of the elevated HI-C background signal between microchromosomes (due to their central position in the nucleus), genome alignments with reference species and mapping of protein coding genes. This process is slow and laborious and there is a high likelihood of sequence content being missed from the assembled chromosomes.

Here, to aid manual curation of bird genomes, we took advantage of conserved gene content to identify dot microchromosome fragments in draft genome assemblies. Using 11 high-quality, manually curated bird genomes generated as part of the VGP, 25 Genomes Project and DToL [4], as well as a near telomere-to-telomere (T2T) assembly of chicken [11], we identified a set of conserved dot microchromosome protiens and have developed MicroFinder [29], a pipeline that uses this protein set to find candidate dot microchromosome contigs from draft assemblies to improve the speed and accuracy of manual curation. Using this approach, we revisited 12 previously released bird genome assemblies and improved the content and representation of their assembled dot microchromosomes.

Findings

Identification of conserved microchromosome proteins

Given the gene-dense nature of microchromosomes and their conserved synteny across birds, we hypothesised that a dense marker set of protein coding genes would enable the identification of microchromosome fragments in draft genome assemblies. To generate a set of marker proteins, we made use of expert-curated genome assemblies generated for the VGP, DToL and 25 Genomes projects. We selected 11 published genome assemblies with NCBI RefSeq or Ensemble rapid release gene-sets (**Supplementary Table 2**). We also included a recent, near-T2T assembly of chicken [11]. Together, these 12 assemblies span nine bird orders and 11 families (**Supplementary Table 2**, **Figure 2A**). Although this is a relatively low proportion of described bird orders (~20%) we cover deep splits in the avian phylogeny [30] with representatives from Galloanserae and Neoaves. Of note, this collection includes three high confidence genome assemblies (bCucCan1, bTaeGut1 and GGswu, herein referred to as the *ToL reference set*) that have undergone extensive manual curation and are commonly used by genome curators at the Welcome Sanger Tree of Life (ToL) program as references for whole genome alignments when curating new bird assemblies. Additionally, six of the selected assemblies have been confirmed to be karyotype-complete based on cytology (**Supplementary Table 2**, **Figure 2A**). Of the remaining assemblies, two species do not have published karyotypes and four likely have missing chromosomes based on expectations from cytology, further highlighting the challenges of generating karyotype-complete genome assemblies for birds even when high-quality data is available and substantial manual curation time has been invested.

To identify conserved, low copy number proteins to use as markers we clustered proteomes from the 12 bird reference genomes into orthogroups (gene families) with OrthoFinder [31,32] and used KinFin [33] to select broadly conserved “fuzzy” orthogroups that have relaxed conservation and copy number constraints (<= 3 copies per species and present in at least 50% of species). In total, 197,759 proteins were clustered into 16,589 orthogroups, of which 9,400 were conserved and single-copy in all species and 14,514 were identified by KinFin as “fuzzy” orthogroups (**Supplementary Table 3** and **Supplementary Data**). We further filtered the KinFin orthogroup set to only include proteins located on dot microchromosomes in any of the three ToL reference species, using the near-T2T GGswu chicken assembly to classify dot chromosome homologs in bCucCan1 and bTaeGut1 (**Figure 2B**-**F**). We reasoned that specifically targeting dot microchromosomes rather than all microchromosomes would be most beneficial for assembly curation as larger microchromosomes are typically much less fragmented than dot microchromosomes. This filtering identified 510 dot microchromosome-associated orthogroups containing 4,510 proteins across all 12 reference species. To reduce redundancy, we clustered the dot microchromosome-associated proteins with CD-HIT [34] to produce a final set containing 2,882 proteins which we refer to as the MicroFinder protein set.

Next, we investigated coverage of MicroFinder loci across the near-T2T GGswu assembly of chicken. The 10 GGswu dot chromosomes have between 15 and 67 GGswu MicroFinder loci per chromosome (307 in total), with an average density of 7.5 loci per Mb of sequence (**Figure 3**). In comparison, the orthoDB10 avian Benchmarking Universal Single-Copy Orthologs (BUSCO) gene set (n = 8,338 orthogroups) has only 3 genes located on dot microchromosomes (**Supplementary Figure 1**), likely due historical difficulties with dot microchromosome assembly leading to severe underrepresentation of genes from these chromosomes in

OrthoDB. Previously, Huang et. al. [11] showed that chicken dot microchromosomes are split into two distinct domains - gene-rich euchromatic regions and repetitive, gene-poor heterochromatic regions, with the euchromatic parts typically occupying a large region of the long arm of each chromosome. In line with this, we find clustering of MicroFinder proteins in high expression, low repeat density regions (**Figure 3**). As such, the high density of MicroFinder proteins in euchromatin will increase the likelihood of identifying genic regions of dot microchromosomes in fragmented genome assemblies.

Protein mapping and assembly ordering to aid genome curation

To make use of the MicroFinder protein set we developed a pipeline to map and count MicroFinder proteins in a draft genome assembly and reorder scaffolds by MicroFinder protein count. This strategy means that putative dot microchromosome scaffolds appear at the beginning of the HI-C contact map separated from other small fragments, enabling curators to quickly identify dot microchromosome content and start building up chromosome-scale scaffolds without having to sift through repetitive unplaced small scaffolds and contigs as is the case for a standard, size-sorted map. The MicroFinder pipeline aligns the MicroFinder protein set to a draft assembly with miniprot [35], selects the top ranking hit for each protein, removes alignments with less than 70% identity and then counts protein alignments per scaffold and outputs a reordered assembly fasta file and associated MicroFinder count data. Optionally, the pipeline can apply a maximum scaffold size cutoff for assembly sorting. During testing we found that macrochromosome scaffolds can sometimes contain a low number of MicroFinder hits, most likely due to the presence of divergent paralogs or mis-mapping. We therefore recommend using a 5 Mb maximum scaffold size cutoff for assembly sorting. Following sorting, new HI-C contact maps can be made for assembly curation in PretextView

[36] using the CurationPretext pipeline [37]. MicroFinder has been packaged up into Docker and Singularity containers for easy deployment [29] and we have developed a training workshop with example datasets to guide users [38].

To demonstrate how MicroFinder can be used as a curation aid, we applied it to the draft (pre curation) DToL genome assembly of *Anas acuta* [39]. MicroFinder identified 61 putative dot chromosome scaffolds shorter than 5 Mb and moved them to the start of the HI-C contact map (**Figure 4**). These scaffolds were manually ordered and rearranged to form 10 chromosomal elements using the gene-rich MicroFinder-identified scaffolds as anchors to build up dot microchromosome models. Applying MicroFinder before manual curation therefore speeds up the curation process by removing the need for curators to trawl through repetitive unplaced small scaffolds and contigs and reduces the risk of small gene-rich dot microchromosome contigs being missed from chromosome models. Furthermore, the pipeline is relatively lightweight and does not require large compute resources. For the *A. acuta* example, MicroFinder used 565 seconds of CPU time across eight cores and consumed a maximum of 8.1 Gb of RAM.

Reassembly of DToL bird genomes using MicroFinder-aided curation

Next, we investigated whether MicroFinder could be used to improve previously released chromosome-scale bird genome assemblies. We ran MicroFinder on 12 DToL bird genome assemblies that had been assembled using PacBio HiFi and HI-C and subjected to manual curation by the ToL curation team (**Supplementary Table 4**). For each assembly, we ran MicroFinder with a 5 Mb maximum scaffold length cutoff and generated a new HI-C contact map for curation in PretextView using the original sequence data. MicroFinder identified between 22 and 74 (mean = 49) putative unplaced dot microchromosome scaffolds per

assembly (**Figure 5a**). We were able to unambiguously place MicroFinder scaffolds onto dot chromosome models in 11 out of 12 of the assemblies, placing between 2 and 16 scaffolds and increasing the total length of assembled chromosomes in 9 out of 12 assemblies, placing between 216 Kb and 4.3 MB of additional content into chromosome models per assembly (average = 1.4 Mb) (**Figure 5b**). Two assemblies, *Accipiter gentilis* (bAccGen1.1) and *Netta rufina* (bNetRuf1.1), had a decrease in assembled chromosome length due to identification of errors in the original assembly. In *A. gentilis*, a large section of repetitive content on chromosome 35 from the start of the chromosome to ~9.6 Mb had been incorrectly joined at a telomere and was moved back to the unplaced assembly content. In *N. rufina*, an unlocalised sequence assigned to chromosome 38 did not have elevated HI-C background signal with this chromosome compared to the others and was also moved back to the unplaced assembly content. In total, MicroFinder enabled the placement of an additional 12.5 MB of dot microchromosome content across 9 DToL genomes. Furthermore, in the case of *Anas acuta* (bAnaAcu1.1), we were able to identify an additional dot microchromosome model that had been missed in the original curation (**Figure 5c**). Unplaceable scaffolds either had ambiguous HI-C signal or were too small to place, reflecting the fragmented nature of dot microchromosome assemblies (**Figure 5c**).

To assess the validity of the MicroFinder-enabled curation of the DToL assemblies we inspected high resolution HI-C contact maps before and after curation (**Supplementary Figures 2 – 12**). Additionally, for each species, we aligned the original assembly and the MicroFinder-updated assembly to the near-T2T chicken assembly and calculated alignment coverage of chicken dot microchromosomes. For assemblies where content had been added to dot microchromosome models, alignment coverage increased by an average of 178 Kb,

ranging from 6 Kb for bGulAri2 to 583 Kb for bGruGru1 (**Supplementarty Figure 13**; **Supplementary Table S5**), indicating that genuine additional dot microchromosome content has been incorporated into the revised assemblies.

Conclusion

Here, we have identified a set of broadly conserved proteins located on the smallest bird microchromosomes, known as dot microchromosomes, and developed a pipeline (MicroFinder) to identify and order putative dot microchromosome scaffolds in draft genome assemblies. By using "fuzzy" orthogroup selection, the MicroFinder protein set includes a large number of broadly conserved single-copy (or low copy number) proteins and provides good coverage across all avian dot microchromosomes (**Figure 3**). Using this strategy, MicroFinder can detect putative dot microchromosome scaffolds in fragmented draft genome assemblies and is an effective curation aid for bird genome assembly, even enabling improvement to genome assemblies that have already undergone expert curation (**Figure 5**). Previously, an integrative method that uses a BAC panel to identify chromosome-specific regions was developed to resolve fragmented assemblies, including identification of microchromosomes [40], however it requires expertise in molecular cytogenetics and is time-consuming and impractical for current large-scale sequencing projects. Instead, MicroFinder provides a quick and easy pipeline to effectively pull-out putative dot microchromosome fragments *in silico*. Furthermore, the MicroFinder approach may be applicable to other systems which have conserved but hard to assemble chromosomes, such as the dot chromosome (Muller element F) in Diptera.

Recently, near-T2T assemblies have been released for chicken, bustard and mallard [11,41,42]. These assemblies achieved higher microchromosome contiguity through the inclusion of

Oxford Nanopore ultra long reads. This approach represents a promising avenue to further improve bird genome assembly quality. However, due to scale and inertia, many projects still rely primarily on PacBio HiFi *de novo* assembly and will greatly benefit from our approach. We recommend MicroFinder is incorporated into bird genome assembly pipelines prior to manual curation to maximise the completeness of dot microchromosome assemblies.

Methods

Meta-analysis of bird karyotype and genome assembly chromosome counts

Genomes on a Tree (GoaT) [43] was used to retrieve bird chromosome counts based on cytology and from chromosome-level assemblies hosted INDC databases (**Supplementary Table 3**). We searched the GOAT database for bird (Aves) species using the "taxon" index of the database, and we excluded species that do not have directly estimated chromosome counts from cytology or that do not have a chromosome-scale genome assembly, retaining 105 species for downstream analysis. For chromosome counts based on genome assemblies, a single summary value was used as the representative chromosome count per species. For each assembly, the chromosome count corresponds to the number of chromosomes identified by the original submitter in the primary assembly (as opposed to the alternate assembly for a taxon). When multiple assemblies were available per taxon, the summary corresponds to the primary haplotype of NCBI RefSeq assembly. Haploid cytology-based chromosome numbers were extracted by halving the diploid number from the Bird Chromosome Database [10] and Animal Chromosome Counts Database (Release 1.0.1) [44] during GoaT import. A single summary value per species was calculated as the mode across all reported values per species. The ranges of values within each dataset were manually

checked to ensure the summary values for chromosome number and haploid numbers from cytology were biologically consistent. We found that most of the variation detected within cytological observations corresponded to +-1 chromosome from the summary mode, consistent with reporting of different total number of chromosomes in different sexes and/or small miscounting from older manuscripts (eg. Makino *et. al.* [45]). The outliers were also manually checked on the original source, and all 7 detected cases corresponded to problematic values in their respective databases; because these values were not used as summaries, they were not included in our meta-analysis, and did not create bias in the data on **Figure 1a**.

Dot chromosome homology assignment between chicken, zebra finch and cuckoo

Pairwise whole genome alignments were carried out between chicken (GGswu), zebra finch (bTaeGut1) and cuckoo (bCucCan1) (**Supplementary Table 2**) using nucmer v4.0.0rc1 [46] and visualised with Dot [47]. Using these alignments, we identified homologs to GGswu dot microchromosomes previously classified by Huang *et. al.* [11].

Orthogroup clustering and identification of the MicroFinder protein set

To identify a set of conserved protein coding genes to use as dot microchromosome markers we built orthogroups across representative bird genome assemblies. We selected 11 published chromosome-scale bird genome assemblies that had NCBI RefSeq or Ensembl rapid release gene-sets and combined them with a recent, near-T2T assembly of chicken [11] (**Supplementary Table 2**). For each species, we selected the longest transcript per gene to be the representative transcript and clustered protein sequences with OrthoFinder v2.5.4 [31,32] in multiple sequence alignment mode (“-M msa”). The resulting orthogroups were

filtered with KinFin v1.1.1 [33] with the parameters "--max 3 -x 0.5" to identify orthogroups present in at least 50 percent of species with a maximum of three gene copies per species. To create the MicroFinder protein set, the KinFin orthogroups were filtered to retain only those with a copy on chicken (GGswu), zebra finch (bTaeGut1) or cuckoo (bCucCan1) dot microchromosomes. Proteins from the filtered orthogroups were then clustered with CD-HIT v4.8.1 [34] using default settings to reduce redundancy.

Phylogenetic analysis

To place the 12 bird reference genomes used to generate the MicroFinder protein set in evolutionary context we carried out phylogenetic analysis using protein sequence alignments generated by OrthoFinder for 9,400 strictly conserved single-copy orthogroups. IQTree v2.3.4 was used to identify the optimal partitioning scheme, carry out model selection, estimate the maximum likelihood phylogeny and carry out 1,000 ultrafast bootstrap replicates to assess tree support [48–52]. The IQTree phylogeny was rooted on the branch leading to Galloanserae (Galliformes plus Anseriformes) following Prum *et. al.* [30].

The MicroFinder pipeline

All steps of the MicroFinder pipeline are implemented in a bash script and the whole pipeline is available as a docker or singularity container [29]. First, the MicroFinder protein set is aligned to the draft genome assembly with miniprot v0.14 [35] with default settings. From the resulting alignments, we retain the top hit and discard alignments with less than 70% identity. MicroFinder protein hits are counted for each scaffold and the input assembly fasta file is sorted by the alignment count. Optionally, a maximum scaffold length cutoff can be applied to the assembly sorting step. MicroFinder outputs a fasta file of the draft assembly sorted by

MicroFinder protein alignment counts, a table of alignment counts per input scaffold and a GFF file of the miniprot alignments. It should be noted that MicroFinder counts reflect the number of protein hits from the MicroFinder protein set rather than counts of individual loci. We opted to map all proteins to maximise sensitivity to detect candidate dot microchromosome scaffolds across a wide range of bird species. The MicroFinder-sorted assembly file should be prepared for manual curation in PretextView [36] with the CurationPretext pipeline [37] with the "--no-sort" parameter used to retain the order of the MicroFinder assembly file in the HI-C contact map. Manual curation of the MicroFinder-ordered HI-C contact map can then proceed following the principles and procedures set out in Howe et. al. [5]

MicroFinder protein distribution in chicken (GGswu) and associated features

We investigated the distribution of MicroFinder proteins across the near-T2T GGswu chicken assembly [11]. MicroFinder protein coordinates were extracted from the GGswu annotation GFF file. To place MicroFinder proteins in context we also estimated genome-wide repeat content and gene expression levels. RNA-seq from a from female chicken liver (SRR18788805) was aligned to the GGswu assembly with HISAT2 v2.2.1 [53] and we calculated read depth in 10 Kb fixed windows using Sambamba v0.8.2 [54]. To estimate repeat density across the GGswu dot chromosomes, we ran RepeatMasker v4.1.8 [55,56] using a manually curated avian repeat library [37,57,58] and calculated repeat density in 10 Kb fixed windows with bedtools coverage v2.31.1 [59] using the RepeatMasker GFF file as input. To compare the distribution of MicroFinder proteins to BUSCO genes we ran BUSCO v5.8.2 [60,61] with the Aves OrthoDB gene set (n = 8338) on the GGswu assembly and extracted the coordinates of BUSCOs located on the dot microchromosomes.

Reassembly of DToL bird genomes with MicroFinder-enabled curation

We selected 12 previously published DToL bird genome assemblies for re-curation with MicroFinder (**Supplementary Table 4**). For each assembly, we ran MicroFinder with a 5 Mb maximum scaffold length cutoff and generated a new HI-C contact map for curation in PretextView using the CurationPretext pipeline v1.0.1 [37] with the "--no-sort" parameter. CurationPretext was provided with the original HI-C and PacBio long reads for each assembly to create a HI-C contact map with read coverage, gap, telomere and simple repeat density tracks. Manual curation [5] was carried out using PretextView v1.0.0 [36]. Following manual curation of each assembly, an AGP file was exported from PretextView and an updated assembly fasta file was generated using pretext-to-asm [62]. To check the validity of changes made during MicroFinder-enabled manual curation, we generated new HI-C contact maps for each species and compared whole genome alignment coverage of dot microchromosomes in the near-T2T GGswu chicken assembly before and after MicroFinder-enabled curation. HI-C contact maps for the revised genome assemblies were generated with CurationPretext using the same sequence data and parameters as for the original MicroFinder-ordered assemblies. For the alignment coverage analysis, the original and MicroFinder-curated assemblies for each species were aligned to the GGswu chicken assembly with minimap2 v2.27-r1193 [63] using the following parameters "-x asm20 --secondary=no". The resulting paf files were converted to bed format and filtered to remove alignments involving unplaced assembly content (i.e. content not in chromosome models in the original or MicroFinder-curated assemblies). We then calculated per-base alignment coverage of the GGswu dot microchromosomes (chromosomes 16 and 29 – 37 [11]) using bedtools coverage v2.31.1 [59] with default settings.

Availability of Source Code and Requirements

Project name: MicroFinder

Project homepage: https://github.com/sanger-tol/MicroFinder

Operating system: Linux / MacOS

Programming language: Ruby / BASH / Docker

Other requirements: None

License: MIT license

RRID: SCR_028196

Data availability

Supplementary data containing OrthoFinder results, the MicroFinder gene set and the 12 re-curated bird genome assemblies is available from Zenodo [64]. For each of the re-curated genome assemblies, we have provided a MicroFinder-ordered HI-C contact map of the original assembly, PretextView savestate and agp files to show changes made to the original assembly, an updated FASTA file of the assembly and a new HI-C contact map of the revised assembly. Mathers et. al. [38] provides a practical guide for using MicroFinder-ordered assemblies for curation with example datasets.

Acknowledgments

We thank Prof. Alex Suh and Dr Valentina Peona for providing access to their curated avian repeat library. We thank Dr Kerstin Howe and Kr Kamil Joran for comments on an earlier version of the manuscript. This work was supported by Wellcome through core funding to the Wellcome Sanger Institute (220540) and the Darwin Tree of Life Discretionary Award

(218328).

Figure legends

Figure 1: Bird genome assemblies are often not karyotype-complete and require extensive manual curation. (**A**) Correspondence analysis of chromosome counts in chromosome-scale genome assemblies versus their respective haploid karyotype for 105 bird species. Colour gradient reflects the number of species in each category (bin of assembly (x) versus karyotype (y) count). The Solid black line marks the match of the chromosome number in assemblies (y-axis) and predicted chromosome number using cytology (x-axis). The dashed diagonal lines indicate ± 1 chromosome margin of error to account for expected variation from assemblies of males (homogametic sex will usually have 1 less assembled chromosome). (**B**) HI-C contact map for the draft genome assembly of *Anser brachyrhynchus* (assembled by the Darwin Tree of Life (DToL) project [Lopez Colom & O'Brien, 2024]). Coloured squares highlight 28 clear chromosomal elements identified during an initial assembly curation (painted "Scaffolds" in PretextView). (**C**) A zoomed in view of the unplaced assembly content grouped at the bottom-right of image (**B**). (**D**) HI-C contact map of the curated *A. brachyrhynchus* genome assembly zoomed in on the smallest 11 chromosomes. Content to the right of the red arrow is unplaced content. Microchromosomes have elevated background HI-C signal but appear as independent elements in the HI-C map.

Figure 2: Phylogeny of annotated chromosome-scale bird reference genomes used to generate the MicroFinder protein set and conserved macro synteny of bird dot microchromosomes. (**A**) Maximum likelihood phylogeny based on a concatenated alignment

of 9,400 conserved single-copy orthogroups. Branch lengths are in amino acid substitutions per site. All nodes have ≥ 99% bootstrap support (1000 ultrafast bootstrap replicates). Species with genome assemblies confirmed to be karyotype-complete based on cytology are highlighted in green. Full details of all assemblies are given in **Supplementary Table 2.** PhyloPic [65] silhouettes of each species are shown at the tree tips. Species marked with an "*" form the ToL reference set and are routinely used as references when assembling diverse bird genomes. (**B** - **F**) Dot mcrochromosome synteny between genomes in the ToL reference set based on whole genome alignments. **F** summarises dot microchromosome homology between chicken (GGswu), zebra finch (bTaeGut1) and cuckoo (bCucCan1) based on the alignments shown in **B** - **E**.

Figure 3: Distribution of MicroFinder proteins on chicken (GGswu) dot microchromosomes. Panels from top to bottom show the location of MicroFinder loci (coral), RNA-seq alignment counts from female chicken liver (SRR18788805) (green) in 10 Kb fixed windows, and transposable element density in 10 Kb fixed windows (blue). To aid visualisation of lower coverage genes, maximum RNAseq read coverage was capped at 25x.

Figure 4: MicroFinder-enabled manual curation of bird dot microchromosomes. Main panel shows HI-C contact map of the MicroFinder-ordered draft (pre curation) genome assembly of *Anas acuta* [39]. *Central panel* shows a zoomed in view of the putative dot microchromosome content that has been moved to the start of the of the assembly by MicroFinder for curation. *Right hand panel* shows zoomed in view of the curated dot microchromosomes.

Figure 5: MicroFinder-enabled re-curation of 12 previously released DToL bird genome assemblies. (**A**) *Bar chart* showing counts of previously unplaced content identified by MicroFinder for 12 genome assemblies. Bars are coloured by whether the scaffolds were placed onto chromosome models during manual curation. (**B**) As for (**A**) but showing total sequence content added to chromosome models during manual curation of the MicroFinder sorted genome assemblies. (**C**) HI-C contact map of the *Anas acuta* genome assembly (bAnaAcu1.1). The figure shows a zoomed in view of the smallest seven chromosomes. Scaffolds in the original assembly are separated by grey lines. Coloured squares indicate "painted" chromosomes and are assigned super scaffold IDs (Scaffold_(n)) by PretextView (shown above each square). Red vertical arrows indicate scaffolds that have been incorporated into chromosome models following MicroFinder-enabled manual re-curation. Scaffold_35 is a chromosome model that was unidentifiable in the original curation. Full stats for all 12 re-curated genome assemblies are provided in **Supplementary Table 4**.

References

1. Rhie A, McCarthy SA, Fedrigo O, Damas J, Formenti G, Koren S, et al.. Towards complete and error-free genome assemblies of all vertebrate species. *Nature*. Nature Research; 2021; doi: 10.1038/s41586-021-03451-0.

2. Feron R, Waterhouse RM. Assessing species coverage and assembly quality of rapidly accumulating sequenced genomes. *Gigascience*. Oxford University Press; 2022; doi: 10.1093/gigascience/giac006.

3. Lewin HA, Robinson GE, Kress WJ, Baker WJ, Coddington J, Crandall KA, et al.. Earth BioGenome Project: Sequencing life for the future of life. *Royal Botanic Gardens*. PNAS; 2001; doi: 10.1073/pnas.1720115115/-/DCSupplemental.

4. The Darwin Tree of Life Project Consortium. Sequence locally, think globally: The Darwin Tree of Life Project. *Proceedings of the National Academy of Sciences*. 2021; doi: 10.1073/pnas.2115642118/-/DCSupplemental.

5. Howe K, Chow W, Collins J, Pelan S, Pointon DL, Sims Y, et al.. Significantly improving the quality of genome assemblies through curation. *Gigascience*. Oxford University Press; 2021; doi: 10.1093/gigascience/giaa153.

6. Lawniczak MKN, Durbin R, Flicek P, Lindblad-Toh K, Wei X, Archibald JM, et al.. Standards recommendations for the Earth BioGenome Project. *PNAS*. 2022; doi: https://doi.org/10.1073/pnas.2115639118.

7. Li H, Durbin R. Genome assembly in the telomere-to-telomere era. Nat. Rev. Genet. Nature Research;

8. Tegelström H, Ryttman H. Chromosomes in birds (Aves): evolutionary implications of macro-and microchromosome numbers and lengths. *Hereditas*. 1981; doi: 10.1111/j.1601-5223.1981.tb01757.x.

9. Griffin DK, Robertson LBW, Tempest HG, Skinner BM. The evolution of the avian genome as revealed by comparative molecular cytogenetics. *Cytogenet Genome Res*. 117:64–772007;

10. Degrandi TM, Barcellos SA, Costa AL, Garnero ADV, Hass I, Gunski RJ. Introducing the Bird Chromosome Database: An Overview of Cytogenetic Studies in Birds. *Cytogenet Genome Res*. S. Karger AG; 2020; doi: 10.1159/000507768.

11. Huang Z, Xu Z, Bai H, Huang Y, Kang N, Ding X, et al.. Evolutionary analysis of a complete chicken genome. *Proc Natl Acad Sci U S A*. National Academy of Sciences; 2023; doi: 10.1073/pnas.2216641120.

12. Newcomer EH. The mitotic chromosomes of the domestic fowl. *Journal of Heredity*. 48:227–341957;

13. Newcomer EH. Accessory chromosomes in the domestic fowl. *Genetics*. 401955;

14. Waters PD, Patel HR, Ruiz-Herrera A, ıa Alvarez-Gonz alez L, Lister NC, Simakov O, et al.. Microchromosomes are building blocks of bird, reptile, and mammal chromosomes. *Proceedings of the National Academy of Sciences*. 2021; doi: https://doi.org/10.1073/pnas.2112494118.

15. van Brink JM. L'expression morphologique de la digamétie chez les sauropsidés et les monotrèmes. *Chromosoma*. 1959; doi: 10.1007/BF00396564.

16. Liu J, Wang Z, Li J, Xu L, Liu J, Feng S, et al.. A new emu genome illuminates the evolution of genome configuration and nuclear architecture of avian chromosomes. *Genome Res*. Cold Spring Harbor Laboratory Press; 2021; doi: 10.1101/GR.271569.120.

17. McQueen HA, Siriaco G, Bird AP. Chicken Microchromosomes Are Hyperacetylated, Early Replicating, and Gene Rich. *Genome Res*. 1998; doi: doi:10.1101/gr.8.6.621.

18. Smith J, Bruley CK, Paton IR, Dunn I, Jones CT, Windsor D, et al.. Differences in gene density on chicken macrochromosomes and microchromosomes. *Anim Genet*. 2000; doi: 10.1046/j.1365-2052.2000.00565.x.

19. Perry BW, Schield DR, Adams RH, Castoe TA. Microchromosomes Exhibit Distinct Features of Vertebrate Chromosome Structure and Function with Underappreciated Ramifications for Genome Evolution. *Mol Biol Evol*. Oxford University Press; 2021; doi: 10.1093/molbev/msaa253.

20. O'Connor RE, Kiazim L, Skinner B, Fonseka G, Joseph S, Jennings R, et al.. Patterns of microchromosome organization remain highly conserved throughout avian evolution. *Chromosoma*. Springer Science and Business Media Deutschland GmbH; 2019; doi: 10.1007/s00412-018-0685-6.

21. Habermann FA, Cremer M, Walter J, Kreth G, Von Hase J, Bauer K, et al.. Arrangements of macro-and microchromosomes in chicken cells. *Chromosome Research*. 9:569–842001;

22. Larivière D, Abueg L, Brajuka N, Gallardo-Alba C, Grüning B, Ko BJ, et al.. Scalable, accessible and reproducible reference genome assembly and evaluation in Galaxy. Nat. Biotechnol. Nature Research;

23. Peona V, Blom MPK, Xu L, Burri R, Sullivan S, Bunikis I, et al.. Identifying the causes and consequences of assembly gaps using a multiplatform genome assembly of a bird-of-paradise. *Mol Ecol Resour*. Blackwell Publishing Ltd; 2021; doi: 10.1111/1755-0998.13252.

24. Barros CP, Derks MFL, Mohr J, Wood BJ, Crooijmans RPMA, Megens HJ, et al.. A new haplotype-resolved turkey genome to enable turkey genetics and genomics research. *Gigascience*. Oxford University Press; 2023; doi: 10.1093/gigascience/giad051.

25. Li M, Sun C, Xu N, Bian P, Tian X, Wang X, et al.. De Novo Assembly of 20 Chicken Genomes Reveals the Undetectable Phenomenon for Thousands of Core Genes on Microchromosomes and Subtelomeric Regions. *Mol Biol Evol*. Oxford University Press; 2022; doi: 10.1093/molbev/msac066.

26. Lopez Colom R, O'Brien M. The genome sequence of the pink-footed goose, Anser brachyrhynchus Baillon, 1834. *Wellcome Open Res*. 2024; doi: 10.12688/wellcomeopenres.23194.1.

27. Wójcik E, Smalec E. Description of the Anser anser Goose Karyotype. *Folia biologica (Kraków)*. 55:1–22007;

28. Uno Y, Nishida C, Hata A, Ishishita S, Matsuda Y. Molecular cytogenetic characterization of repetitive sequences comprising centromeric heterochromatin in three Anseriformes species. *PLoS One*. Public Library of Science; 2019; doi: 10.1371/journal.pone.0214028.

29. Mathers TC, Paulini M, Sotero-Caio CG, Wood JMD: MicroFinder. GitHub; https://github.com/sanger-tol/MicroFinder

30. Prum RO, Berv JS, Dornburg A, Field DJ, Townsend JP, Lemmon EM, et al.. A comprehensive phylogeny of birds (Aves) using targeted next-generation DNA sequencing. *Nature*. Nature Publishing Group; 2015; doi: 10.1038/nature15697.

31. Emms DM, Kelly S. OrthoFinder: solving fundamental biases in whole genome comparisons dramatically improves orthogroup inference accuracy. *Genome Biol*. Genome Biology; 2015; doi: 10.1186/s13059-015-0721-2.

32. Emms DM, Kelly S. OrthoFinder: Phylogenetic orthology inference for comparative genomics. *Genome Biol*. Genome Biology; 2019; doi: 10.1186/s13059-019-1832-y.

33. Laetsch DR, Blaxter ML. KinFin: Software for taxon-aware analysis of clustered protein sequences. *G3: Genes, Genomes, Genetics*. Genetics Society of America; 2017; doi: 10.1534/g3.117.300233.

34. Fu L, Niu B, Zhu Z, Wu S, Li W. CD-HIT: Accelerated for clustering the next-generation sequencing data. *Bioinformatics*. 2012; doi: 10.1093/bioinformatics/bts565.

35. Li H. Protein-to-genome alignment with miniprot. *Bioinformatics*. Oxford University Press; 2023; doi: 10.1093/bioinformatics/btad014.

36. PretextView. https://github.com/sanger-tol/PretextView Accessed 2026 Mar 25.

37. Pointon D-LB: sanger-tol/curationpretext. Zenodo; https://doi.org/10.5281/zenodo.14621949 (2025).

38. Mathers TC, Paulini M, Collins J, Absolon D, Pelan S, Wood J: Manual curation of bird microchromosomes with HiC and gene mapping. Zenodo; https://doi.org/10.5281/zenodo.18459675 (2024).
39. O'Brien MF, Lopez Colom R. The genome sequence of the northern pintail, Anas acuta Linnaeus, 1758. *Wellcome Open Res*. 2024; doi: 10.12688/wellcomeopenres.22770.1.
40. Damas J, O'Connor R, Farré M, Lenis VPE, Martell HJ, Mandawala A, et al.. Upgrading short-read animal genome assemblies to chromosome level using comparative genomics and a universal probe set. *Genome Res*. Cold Spring Harbor Laboratory Press; 2017; doi: 10.1101/gr.213660.116.
41. Luo H, Jiang X, Li B, Wu J, Shen J, Xu Z, et al.. A high-quality genome assembly highlights the evolutionary history of the great bustard (Otis tarda, Otidiformes). *Commun Biol*. Nature Research; 2023; doi: 10.1038/s42003-023-05137-x.
42. Hu J, Song L, Ning M, Niu X, Han M, Gao C, et al.. A new chromosome-scale duck genome shows a major histocompatibility complex with several expanded multigene families. *BMC Biol*. BioMed Central Ltd; 2024; doi: 10.1186/s12915-024-01817-0.
43. Challis R, Kumar S, Sotero-Caio C, Brown M, Blaxter M. Genomes on a Tree (GoaT): A versatile, scalable search engine for genomic and sequencing project metadata across the eukaryotic tree of life. *Wellcome Open Res*. F1000 Research Ltd; 2023; doi: 10.12688/wellcomeopenres.18658.1.
44. Román-Palacios C, Medina CA, Zhan SH, Barker MS. Animal chromosome counts reveal a similar range of chromosome numbers but with less polyploidy in animals compared to flowering plants. *J Evol Biol*. John Wiley and Sons Inc; 2021; doi: 10.1111/jeb.13884.
45. Makino S. An atlas of the chromosome numbers in animals. Ames : The Iowa State College Press.;

46. Marçais G, Delcher AL, Phillippy AM, Coston R, Salzberg SL, Zimin A. MUMmer4: A fast and versatile genome alignment system. *PLoS Comput Biol*. 2018; doi: 10.1371/journal.pcbi.1005944.

47. dot. https://github.com/marianattestad/dot Accessed 2026 Mar 25.

48. Minh BQ, Schmidt HA, Chernomor O, Schrempf D, Woodhams MD, Von Haeseler A, et al.. IQ-TREE 2: New models and efficient methods for phylogenetic inference in the genomic era. *Mol Biol Evol*. 2020; doi: 10.1093/molbev/msaa015.

49. Chernomor O, Von Haeseler A, Minh BQ. Terrace Aware Data Structure for Phylogenomic Inference from Supermatrices. *Syst Biol*. Oxford University Press; 2016; doi: 10.1093/sysbio/syw037.

50. Minh BQ, Schmidt HA, Chernomor O, Schrempf D, Woodhams MD, Von Haeseler A, et al.. IQ-TREE 2: New Models and Efficient Methods for Phylogenetic Inference in the Genomic Era. *Mol Biol Evol*. Oxford University Press; 2020; doi: 10.1093/molbev/msaa015.

51. Minh BQ, Dang CC, Vinh LS, Lanfear R. QMaker: Fast and Accurate Method to Estimate Empirical Models of Protein Evolution. *Syst Biol*. Oxford University Press; 2021; doi: 10.1093/sysbio/syab010.

52. Kalyaanamoorthy S, Minh BQ, Wong TKF, Von Haeseler A, Jermiin LS. ModelFinder: Fast model selection for accurate phylogenetic estimates. *Nat Methods*. 2017; doi: 10.1038/nmeth.4285.

53. Kim D, Langmead B, Salzberg SL. HISAT: A fast spliced aligner with low memory requirements. *Nat Methods*. 2015; doi: 10.1038/nmeth.3317.

54. Tarasov A, Vilella AJ, Cuppen E, Nijman IJ, Prins P. Sambamba: Fast processing of NGS alignment formats. *Bioinformatics*. 2015; doi: 10.1093/bioinformatics/btv098.

55. Smit AFA, Hubley R, Green P. RepeatMasker Open-4.0.

56. Tarailo-Graovac M, Chen N. Using RepeatMasker to identify repetitive elements in genomic sequences. *Curr Protoc Bioinformatics*. 2009; doi: 10.1002/0471250953.bi0410s25.

57. Peona V, Palacios-Gimenez OM, Blommaert J, Liu J, Haryoko T, Jønsson KA, et al.. The avian W chromosome is a refugium for endogenous retroviruses with likely effects on female-biased mutational load and genetic incompatibilities. *Philosophical Transactions of the Royal Society B: Biological Sciences*. Royal Society Publishing; 2021; doi: 10.1098/rstb.2020.0186.

58. Peona V, Palacios-Gimenez OM, Lutgen D, Olsen RA, Kakhki NA, Andriopoulos P, et al.. An annotated chromosome-scale reference genome for Eastern black-eared wheatear (Oenanthe melanoleuca). *G3: Genes, Genomes, Genetics*. Genetics Society of America; 2023; doi: 10.1093/g3journal/jkad088.

59. Quinlan AR, Hall IM. BEDTools: A flexible suite of utilities for comparing genomic features. *Bioinformatics*. 2010; doi: 10.1093/bioinformatics/btq033.

60. Waterhouse RM, Seppey M, Simao FA, Manni M, Ioannidis P, Klioutchnikov G, et al.. BUSCO applications from quality assessments to gene prediction and phylogenomics. *Mol Biol Evol*. 2018; doi: 10.1093/molbev/msx319.

61. Simão FA, Waterhouse RM, Ioannidis P, Kriventseva E V., Zdobnov EM. BUSCO: Assessing genome assembly and annotation completeness with single-copy orthologs. *Bioinformatics*. 2015; doi: 10.1093/bioinformatics/btv351.

62. Utilities for Tree of Life AGP and TPF Assembly Files. https://github.com/sanger-tol/agp-tpf-utils Accessed 2026 Mar 25.

63. Li H. Sequence analysis Minimap2: pairwise alignment for nucleotide sequences. *Bioinformatics*. 2018; doi: 10.1093/bioinformatics/bty191.

64. Mathers TC, Paulini M, Sotero-Caio CG, Wood JMD: Supplementary Data for: MicroFinder: Conserved gene-set mapping and assembly ordering for manual curation of bird microchromosomes [Data set]. Zenodo; 10.5281/zenodo.19233780 (2026).

65. Keesey TM: PhyloPic. https://www.phylopic.org/ Accessed 2026 Mar 25.

Figure 1

Click here to access/download;Figure;Figure 1.pdf

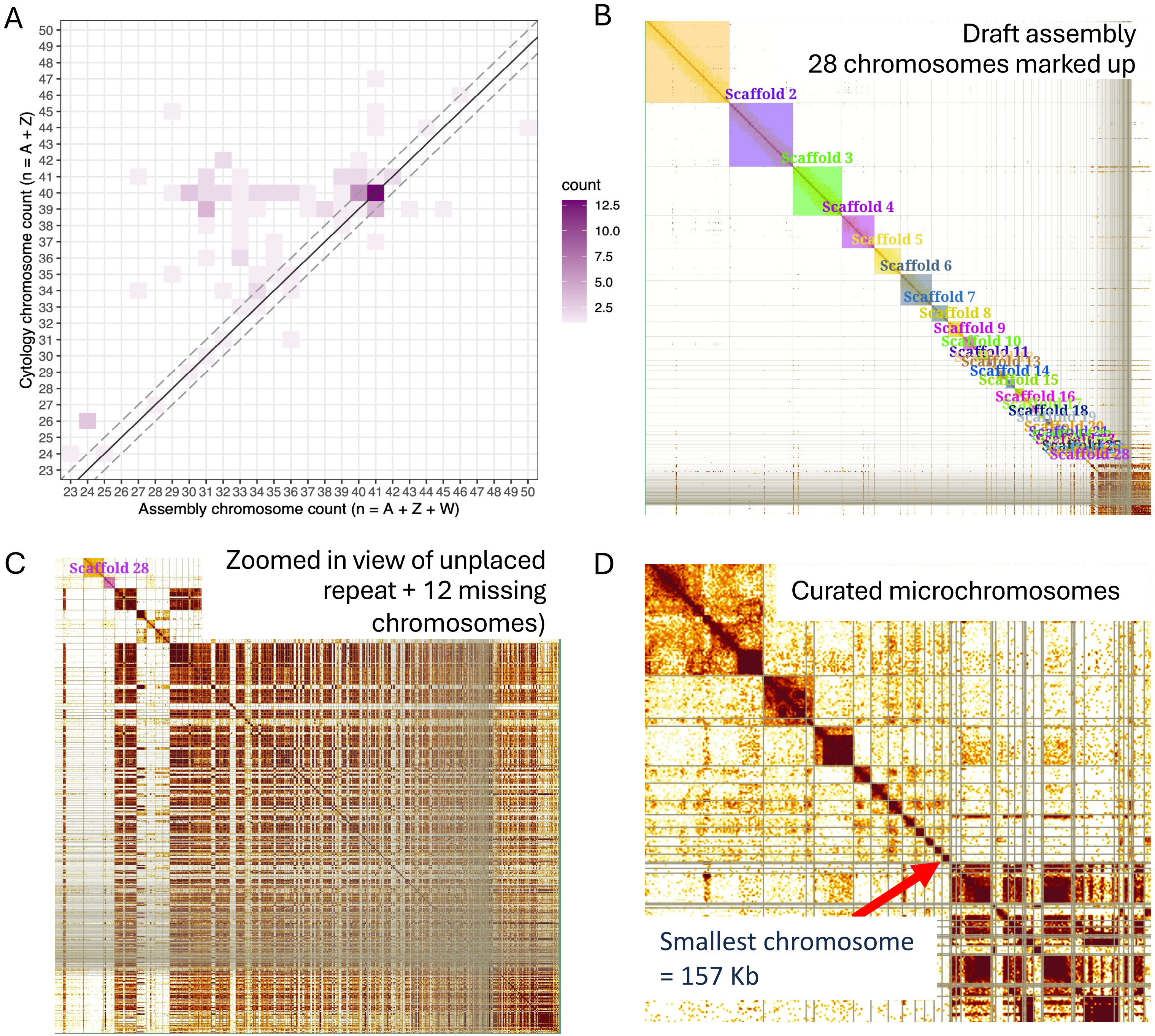

Figure 2

Click here to access/download;Figure;Figure 2.pdf

A

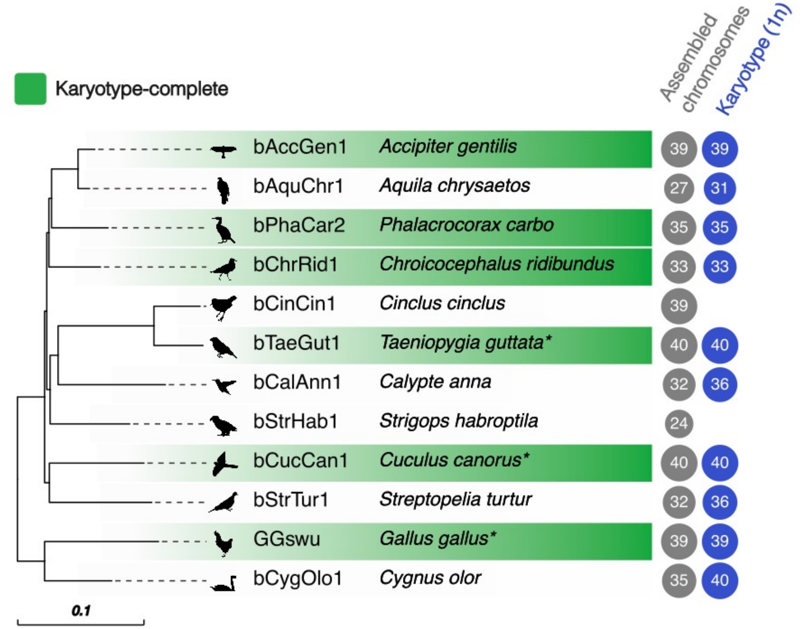

B C

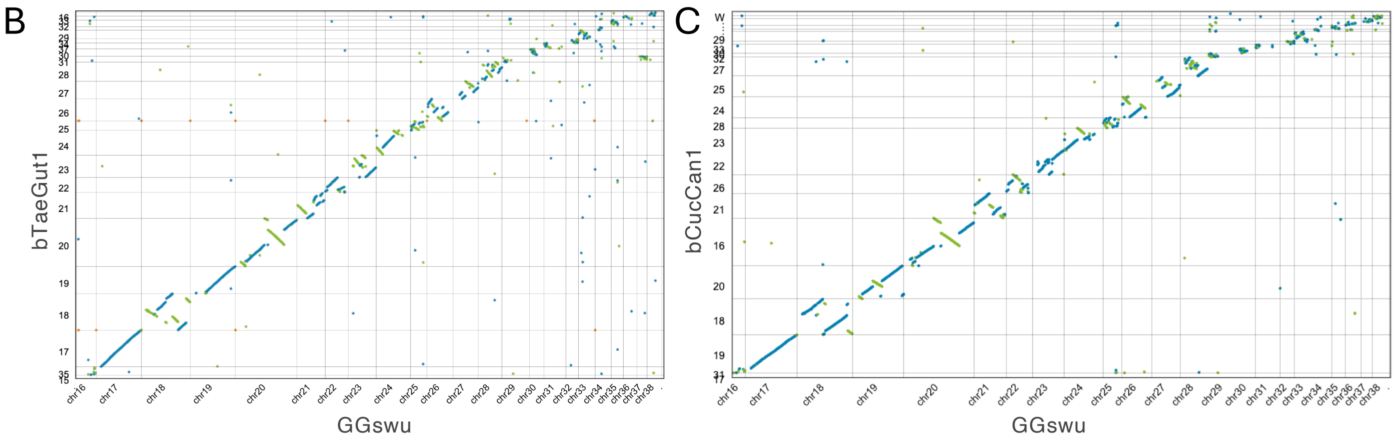

D E

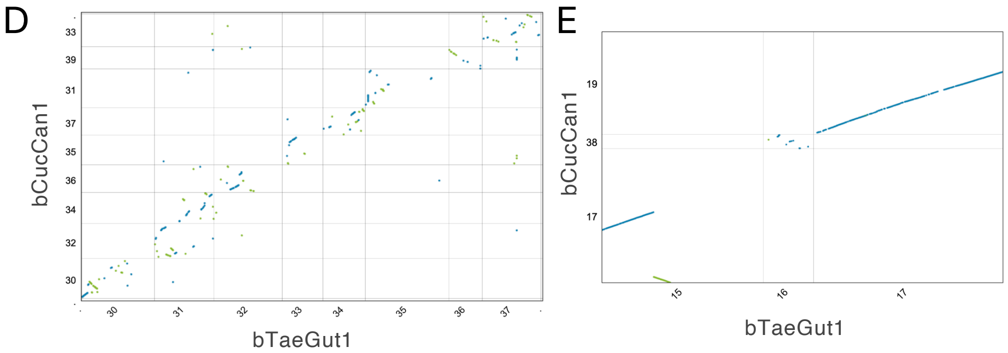

F

GGswu	bTaeGut1	bCucCan1
chr16	35	31
chr29	31	32
chr30	30	30
chr31	37	33
chr32	34	37
chr34	36	39*
chr35	32	36
chr36	33	35*
chr37	31	34
chr38	16	38

*Inferred from bTaeGut1 vs bCucCan1 alignment

Fusion (highlighted)

Figure 3

Click here to access/download;Figure;Figure 3.pdf

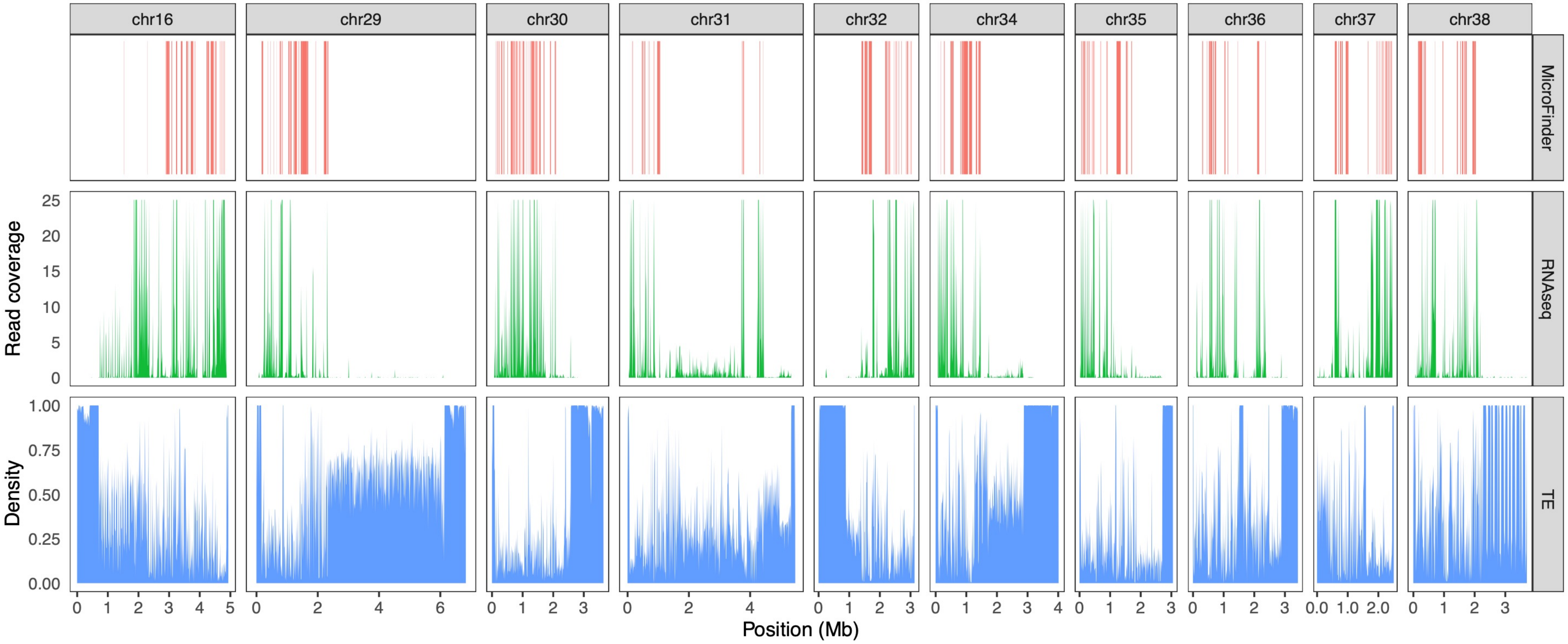

Figure 4

Click here to access/download;Figure;Figure 4.pdf

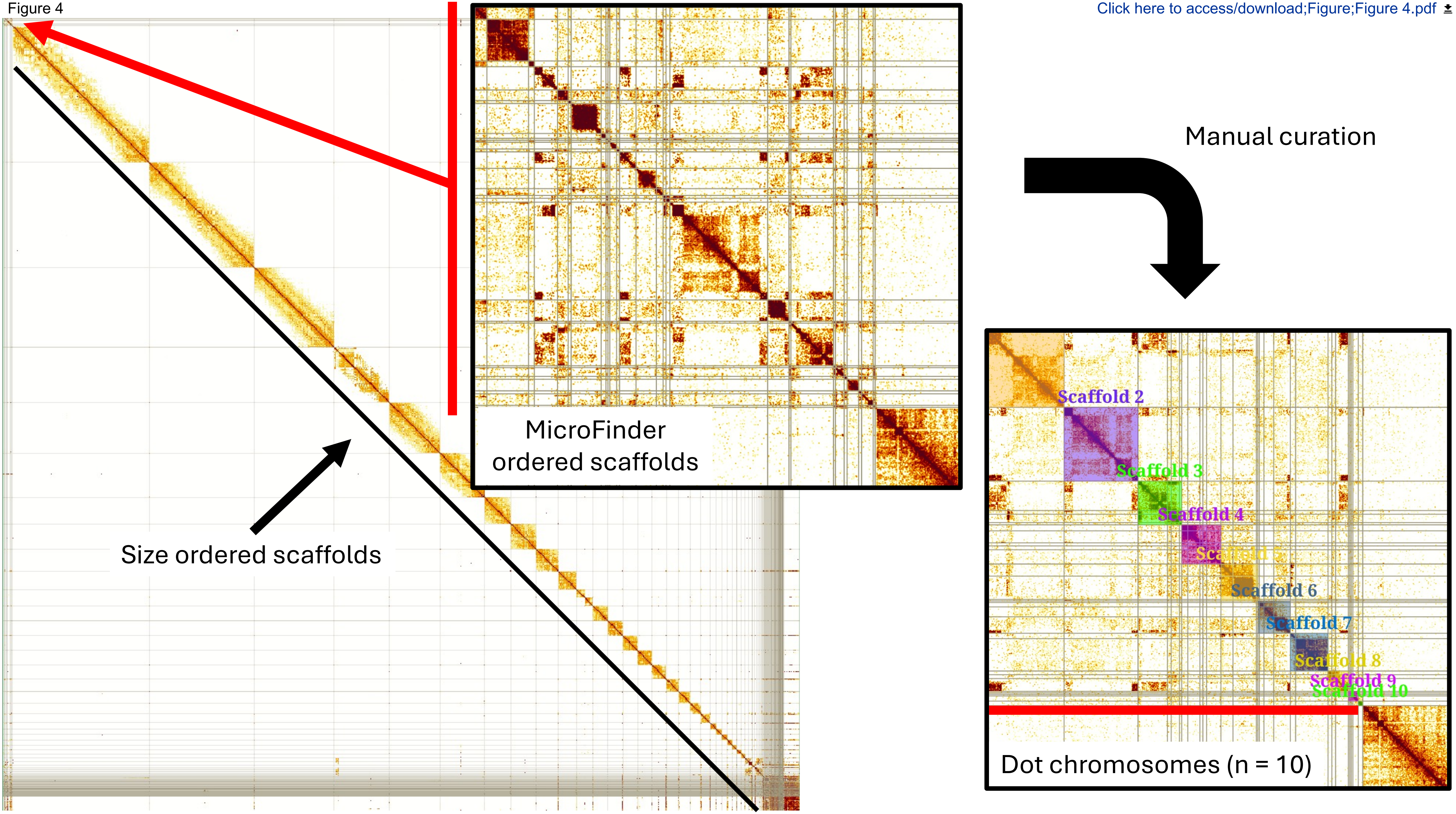

Figure 5

Click here to access/download;Figure;Figure 5.pdf

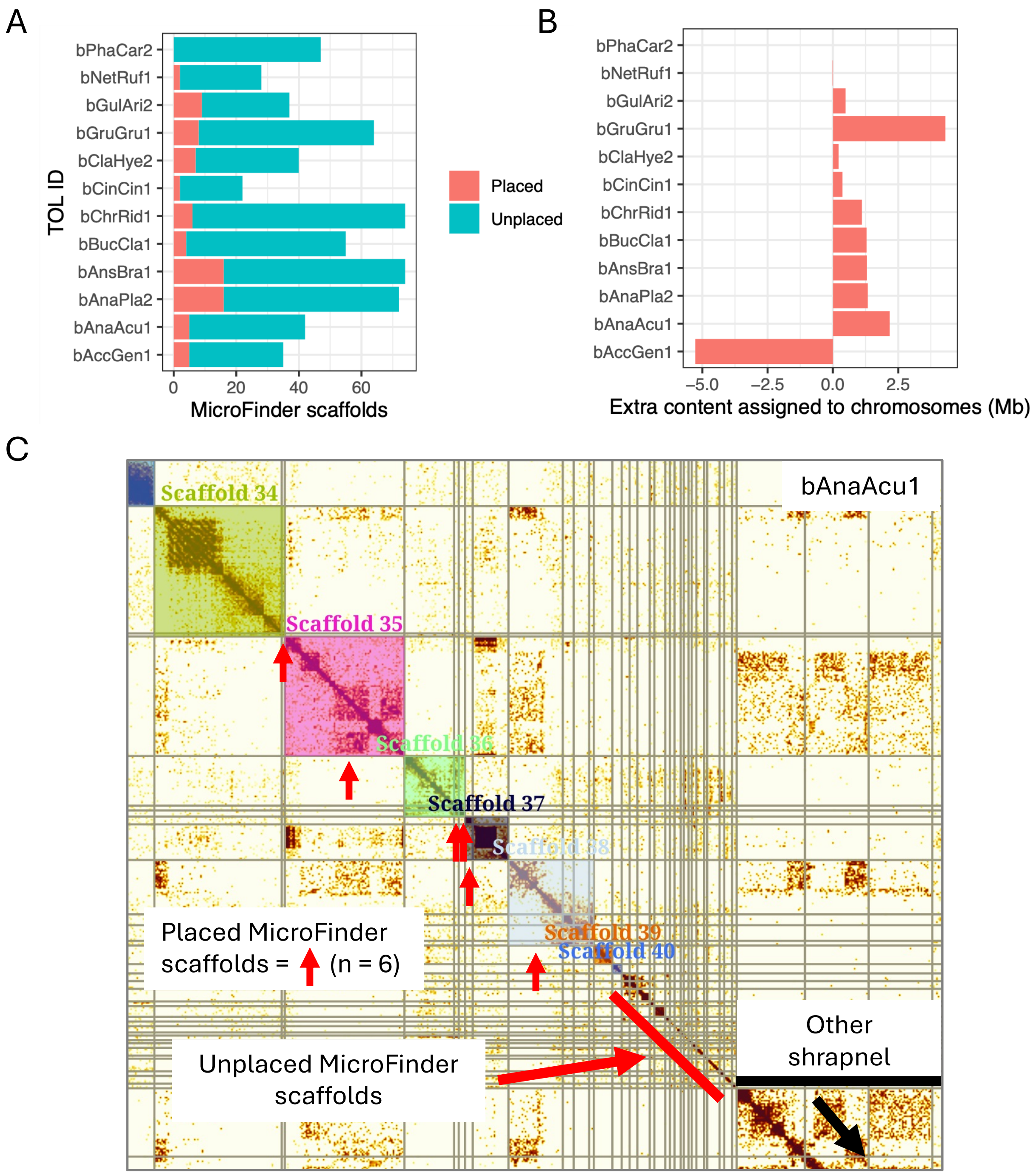

Supplementary Table 1

Click here to access/download
Supplementary Material
Table_S1_GoaT_data.xlsx

Supplementary Table 2

Click here to access/download
Supplementary Material
Table_S2_reference_genomes.xlsx

Supplementary Table 3

Click here to access/download
Supplementary Material
Table_S3_orthofinder_kinfin_microfinder.xlsx

Supplementary Table 4

Click here to access/download
Supplementary Material
Table_S4_recuration.xlsx

Supplementary Table 5

Click here to access/download
Supplementary Material
Table_S5_recuration_align_cov.xlsx

Click here to access/download
Supplementary Material
Supplementary Figures.pdf

Response to reviewer comments

Click here to access/download
Supplementary Material
Response.docx
