# Supplementary material for: MicroFinder: conserved gene-set mapping and assembly ordering for manual curation of bird dot microchromosomes
Source: Gigascience. 2026 Apr 3;15:giag036. doi: 10.1093/gigascience/giag036 (PMC13192246; doi:10.1093/gigascience/giag036)
Supplement: giag036_Reviewer_1_Report_original_submission — Reviewer 1 -- 8/12/2025 [file giag036_reviewer_1_report_original_submission.pdf]

Reviewer Report

Title: MicroFinder: conserved gene-set mapping and assembly ordering for manual curation of bird microchromosomes

Version: Original Submission **Date:** 8/12/2025

Reviewer name: Reviewer 1

Reviewer Comments to Author:

I am very happy to see that MicroFinder is going to be published! Last year I used it very often to curated the bird assemblies.
I found no major issues, but only the minor one.
The only crucial (but still technical issue) is that your protein dataset is from dot microchromosomes, i.e. not from the all microchromosomes.
So I highly recommend to use "dot microchromosomes" where relevant including the title of the manuscript.
Minor issues:
row 19 (Abstract background) change "major goal" to a softer statement. Generation of the assemblies is a very important task of bioiversity genomics but not a major one
row 54-55 Do you imply that typical bird genome contains 37-41 chromosome pairs? There are a lot of birds with lower number of chromosome, so i am not sure that it is typical.. Also a reference to publication from 1981 looks outdated
row 109 - why only eleven assemblies were selected?
row 111 - 112 Please, highlight how many orders/families were not covered
rows 129 - 137 This lines are in some contradiction with all the text including the abstract. Your dataset is focused on a dot chromosomes and not on the all microchromosomes. I suggest to replace "microchromosomes" nearly everywhere to "dot microchromosomes" including the title
row 173 - 185 I am very skeptical about expanding the results obtained on a single genome assembly to the whole family, especially if remember that your dataset covers less than a half of bird orders. My experience with Microfinder tells that sometimes it select contigs/scaffold belonging to macrochromosomes. However, not many and they are usually short. Please, soften statements
row 429 Reference 13 is in French and doesn't have an English translation of the title

Methods

Are the methods appropriate to the aims of the study, are they well described, and are necessary controls included? Choose an item.

Conclusions

Are the conclusions adequately supported by the data shown? Choose an item.

Reporting Standards

Does the manuscript adhere to the journal's guidelines on [minimum standards of reporting?] Choose an item.

Choose an item.

Statistics

Are you able to assess all statistics in the manuscript, including the appropriateness of statistical tests used? Choose an item.

Quality of Written English

Please indicate the quality of language in the manuscript: Choose an item.

Declaration of Competing Interests

Please complete a declaration of competing interests, considering the following questions:

- Have you in the past five years received reimbursements, fees, funding, or salary from an organisation that may in any way gain or lose financially from the publication of this manuscript, either now or in the future?
- Do you hold any stocks or shares in an organisation that may in any way gain or lose financially from the publication of this manuscript, either now or in the future?
- Do you hold or are you currently applying for any patents relating to the content of the manuscript?
- Have you received reimbursements, fees, funding, or salary from an organization that holds or has applied for patents relating to the content of the manuscript?
- Do you have any other financial competing interests?
- Do you have any non-financial competing interests in relation to this paper?

If you can answer no to all of the above, write 'I declare that I have no competing interests' below. If your reply is yes to any, please give details below.

I declare that I have no competing interests

I agree to the open peer review policy of the journal. I understand that my name will be included on my report to the authors and, if the manuscript is accepted for publication, my named report including any attachments I upload will be posted on the website along with the authors' responses. I agree for my report to be made available under an Open Access Creative Commons CC-BY license (http://creativecommons.org/licenses/by/4.0/). I understand that any comments which I do not wish to be included in my named report can be included as confidential comments to the editors, which will not be published.

Choose an item.

To further support our reviewers, we have joined with Publons, where you can gain additional credit to further highlight your hard work (see: https://publons.com/journal/530/gigascience). On publication of this paper, your review will be automatically added to Publons, you can then choose whether or not to claim your Publons credit. I understand this statement.

Yes Choose an item.
