# Supplementary material for: MicroFinder: conserved gene-set mapping and assembly ordering for manual curation of bird dot microchromosomes
Source: Gigascience. 2026 Apr 3;15:giag036. doi: 10.1093/gigascience/giag036 (PMC13192246; doi:10.1093/gigascience/giag036)
Supplement: giag036_Reviewer_2_Report_original_submission — Reviewer 2 -- 9/30/2025 [file giag036_reviewer_2_report_original_submission.pdf]

Reviewer Report

Title: MicroFinder: conserved gene-set mapping and assembly ordering for manual curation of bird microchromosomes

Version: Original Submission **Date:** 9/30/2025

Reviewer name: Reviewer 2

Reviewer Comments to Author:

I had the privilege of reviewing the manuscript titled "MicroFinder: conserved gene-set mapping and assembly ordering for manual insertion of bird microchromosomes" by Mathers et al. The manuscript presents a conserved gene set linked to bird microchromosomes for identifying putative contigs/scaffolds. Subsequently, microchromosomes contigs/scaffolds can be made into their corresponding chromosome models using orthogonal evidence from HiC data. MicroFinder utilises the current knowledge of microchromosome conservation across birds. This approach is similar to assembly evaluation method using BUSO genes.

One of the major limitation of the manuscript is the lack of validation or supportive evidence to show that manual curation results after applying MicroFinder hints are valid and robust. Authors can perform local synteny or chromosome scale alignments analyses and conservation property evaluation to demonstrate that results of assembly curation are valid. Authors can also report metrics of HiC contact maps before and after curation for inter and intra chromosomes contacts to demonstrate improvements. If this is not done, authors may have to remove results and methods corresponding to manual curation so as to focus on genes that are found in "putative" microchromosomes.

Manuscript is generally well written with some minor concerns. Analyses presented are generally robust.

It was confusing to read the difference between micro and dot chromosomes. I encourage authors to avoid "dot" chromosome term. Although it has been used in literature in the past, we can do without that term. There is no strong evidence to suggest if micro and dot chromosomes have any significant functional or system level differences. Best to avoid the term.

If authors insist on using the dot nomenclature, a justification and explanation would be required with clear definitions for both. Also, the name of the workflow may need to change as well. I leave it up to authors to make that call.

Similarly I encourage authors avoid using the term shrapnel for small unplaced contigs. Just use small unplaced contigs instead.

Finding section contains a lot of information that belongs in methods section. For example line numbers 109-117 122-125 135-137 154-156 160-164 167-172 187-192. Please revise the text so that findings section doesn't have any methods description.

A definition of what is a orthogroup and fuzzy orthogroup is required.

Result/findings section needs significant improvements. Authors have relegated much of the results to tables in supplementary information. I insist that authors summarise those results in a meaningful descriptive way and refer to supplementary information for extra details.

Lines 176-177 mentions about the manual curation of micro chromosomes. I would like to see the rules and decisions that were employed to join or break or reorder contigs/scaffolds into a chromosome model.
Authors have mentioned that 216kb-4.3mb of additional content per assembly was added. This is incorrect as the sequence content was already present in the assembly. It is just reorganised into microchromosome scaffolds. Please correct the text to say that unplaced scaffolds are organised into putative microchromosomes.
Lines 108-199 mentions about errors in original assembly. A description about the type of errors would be required.
Authors should discuss the property of eagles, falcons and parrots with rearranged/fused micro chromosomes. The proposed method may not be effective in such instances.
Authors suggest the use of 5Mbp cut off. However, in instances where a micro chromosome is incorrectly placed with a macro- chromosome may miss these instances. Authors discuss this as paralog or misalignment related issues. I suggest that authors provide a metric for the success/failure of identifying genes similar to BUSCO. Authors can run the software on all available bird genomes to define the property of such metric for each gene. Result section can explain proportions of 9400 found on macro vs micro. Proportions of 14k fuzzy genes on micro vs macro, their copy status. 9400 + 14514 doesn't add up to 16,589 orthogroup. Something is not clearly described about those numbers. Please improve the text to make meaningful assessments of conserved gene sets on Microchromosomes for it to be useful for the research community.
Methods:
Lines 233-234: what is taxon in this context? Please clarify. There is also a mention of taxa with missing data. What data were missing? Please clarify.
Lines 236-237: do authors mean that chromosomes identified by the submitter of primary assembly? Please clarify.
For each species, authors should refer to refseq version of the assembly for posterity as well. Common names of species may be useful too for broad readership.
Line 254: please modify the section header to remove assembly version as they are not useful
Methods describing the orthogroup clustering should include details about how alignments were filtered and processed. This is currently missing.
Significance of phylogenetic analyses in the context of manuscript is not very clear. May be remove that section. Perhaps authors can utilise the phylogenetic distance as a way to discuss how conserved gene sets are behaving between species based on distance.
Results section can include run time and compute resource usage metrics for others to estimate resource requirements for such analyses.
Updated assemblies can be submitted to NCBI. Authors should consider this.

Methods

Are the methods appropriate to the aims of the study, are they well described, and are necessary controls included? Choose an item.

Conclusions

Are the conclusions adequately supported by the data shown? Choose an item.

Reporting Standards

Does the manuscript adhere to the journal's guidelines on minimum standards of reporting? Choose an item.

Choose an item.

Statistics

Are you able to assess all statistics in the manuscript, including the appropriateness of statistical tests used? Choose an item.

Quality of Written English

Please indicate the quality of language in the manuscript: Choose an item.

Declaration of Competing Interests

Please complete a declaration of competing interests, considering the following questions:

- Have you in the past five years received reimbursements, fees, funding, or salary from an organisation that may in any way gain or lose financially from the publication of this manuscript, either now or in the future?
- Do you hold any stocks or shares in an organisation that may in any way gain or lose financially from the publication of this manuscript, either now or in the future?
- Do you hold or are you currently applying for any patents relating to the content of the manuscript?
- Have you received reimbursements, fees, funding, or salary from an organization that holds or has applied for patents relating to the content of the manuscript?
- Do you have any other financial competing interests?
- Do you have any non-financial competing interests in relation to this paper?

If you can answer no to all of the above, write 'I declare that I have no competing interests' below. If your reply is yes to any, please give details below.

I declare that I have no competing interests.

I agree to the open peer review policy of the journal. I understand that my name will be included on my report to the authors and, if the manuscript is accepted for publication, my named report including any attachments I upload will be posted on the website along with the authors' responses. I agree for my report to be made available under an Open Access Creative Commons CC-BY license (http://creativecommons.org/licenses/by/4.0/). I understand that any comments which I do not wish to

be included in my named report can be included as confidential comments to the editors, which will not be published.

Choose an item.

To further support our reviewers, we have joined with Publons, where you can gain additional credit to further highlight your hard work (see: https://publons.com/journal/530/gigascience). On publication of this paper, your review will be automatically added to Publons, you can then choose whether or not to claim your Publons credit. I understand this statement.

Yes Choose an item.
